# Overexpression of wild-type human amyloid precursor protein alters GABAergic transmission

**DOI:** 10.1038/s41598-021-97144-3

**Published:** 2021-09-02

**Authors:** Anna Kreis, Jana Desloovere, Nuria Suelves, Nathalie Pierrot, Xavier Yerna, Farah Issa, Olivier Schakman, Roberta Gualdani, Marie de Clippele, Nicolas Tajeddine, Pascal Kienlen-Campard, Robrecht Raedt, Jean-Noël Octave, Philippe Gailly

**Affiliations:** 1 Laboratory of Cell Physiology, Institute of Neuroscience, Université Catholique de Louvain, av. Mounier 53/B1.53.17, 1200 Brussels, Belgium; 2 Faculty of Medicine and Health Sciences, Universiteit Gent, C. Heymanslaan 10, 9000 Gent, Belgium; 3 Alzheimer Dementia Group, Institute of Neuroscience, Université Catholique de Louvain, av. Mounier 53, 1200 Brussels, Belgium

**Keywords:** Inhibition-excitation balance, Cellular neuroscience, Ion channels in the nervous system, Molecular neuroscience, Synaptic plasticity

## Abstract

The function of the amyloid precursor protein (APP) is not fully understood, but its cleavage product amyloid beta (Aβ) together with neurofibrillary tangles constitute the hallmarks of Alzheimer’s disease (AD). Yet, imbalance of excitatory and inhibitory neurotransmission accompanied by loss of synaptic functions, has been reported much earlier and independent of any detectable pathological markers. Recently, soluble APP fragments have been shown to bind to presynaptic GABA_B_ receptors (GABA_B_Rs), subsequently decreasing the probability of neurotransmitter release. In this body of work, we were able to show that overexpression of wild-type human APP in mice (hAPP_wt_) causes early cognitive impairment, neuronal loss, and electrophysiological abnormalities in the absence of amyloid plaques and at very low levels of Aβ. hAPP_wt_ mice exhibited neuronal overexcitation that was evident in EEG and increased long-term potentiation (LTP). Overexpression of hAPP_wt_ did not alter GABAergic/glutamatergic receptor components or GABA production ability. Nonetheless, we detected a decrease of GABA but not glutamate that could be linked to soluble APP fragments, acting on presynaptic GABA_B_Rs and subsequently reducing GABA release. By using a specific presynaptic GABA_B_R antagonist, we were able to rescue hyperexcitation in hAPP_wt_ animals. Our results provide evidence that APP plays a crucial role in regulating inhibitory neurotransmission.

## Introduction

The amyloid precursor protein (APP) is a single-pass transmembrane protein that is highly conserved in the animal kingdom. In humans, the *APP* gene is located on chromosome 21 and its alternative splicing results in three isoforms containing 695, 751 and 770 amino acids, with APP_695_ being predominantly expressed in neurons^1,2^. The physiological function of APP is not fully understood but its metabolism, as a precursor of amyloid β peptides (Aβ), plays a central role in initiating Alzheimer’s disease (AD). APP can be processed in two major pathways: the amyloidogenic and non-amyloidogenic, generating biologically active fragments with different functions. Amyloidogenic processing of APP by β-site APP cleaving enzyme (BACE1) with subsequent cleavage by presenilin-containing γ-secretase, liberates soluble APP beta (sAPPβ) and Aβ^3^. Aβ monomers can further aggregate into soluble oligomers, protofibrils and large insoluble amyloid fibrils that further assemble into amyloid plaques, promoting neurodegeneration constituting a key step in the pathophysiology of AD^4^. In the non-amyloidogenic pathway, APP cleavage by α-secretase (ADAM10) yields soluble APP alpha (sAPPα) preventing the production of Aβ^5^. Over the years, Aβ plaque deposition and the resulting neuronal loss due to the amyloid cascade was the focal point in AD research. However, despite the evidence suggesting a causal role of Aβ in AD, including disease-generating APP mutations, it has been shown early on that synaptic function is affected at the earliest stages of AD and partially independently from the formation of Aβ plaques^6,7^. Moreover, clinical trials using BACE1 or γ-secretase inhibitors to reduce Aβ formation or anti-Aβ antibodies in order to minimize amyloid deposits in AD patients, have been unsuccessful thus far^8–10^. The lack of treatment success of some Aβ-centered medications might be due to the residual presence of Aβ oligomers. Promising strides in Aβ plaque treatment have been made by aducanumab, although its efficacy is still under discussion^11,12^. The function of Aβ in AD becomes even more complex when considering that the binding of Aβ oligomers to synapses and their adverse consequences require the presence of APP^13^. Another disease associated with APP is the Down syndrome (DS). Individuals with DS carry three copies of chromosome 21 resulting in triplication of dosage-sensitive genes that greatly enhance the risk for AD in DS individuals. AD and DS, both neurodegenerative diseases with a common neuropathological basis, have been associated with seizure activity and epilepsy in human subjects^14^ as well as increased excitatory transmission in animal models^15,16^. The partial overlap in the neuropathology of AD and DS suggests that some common pathogenic mechanisms may exist and are related to APP, which is known to regulate the balance between excitatory and inhibitory neurotransmission. Interestingly, it has been shown that APP overexpression, and not the resulting overproduction of Aβ, is responsible for EEG abnormalities such as network hyperexcitability and epileptiform spikes in APP overexpressing mice^17,18^. APP has been associated with neuronal proliferation and differentiation^19,20^ and plays a role in neurite outgrowth as well as synapse formation^21,22^. The manner in which APP affects neurotransmission is a complex question. APP is abundantly expressed in presynaptic boutons where it is a key partner of multiple presynaptic proteins^23^, and its cleavage products affect both excitatory^24,25^ and inhibitory^26,27^ synaptic transmission. Glutamate is the main excitatory neurotransmitter in the CNS that contributes to memory, learning, and synaptic plasticity^28,29^. Glutamate exerts its excitatory function via the N-methyl-D-aspartate receptor (NMDAR) and α-amino-3-hydroxy-5-methyl-4-isoxazolepropionic acid receptor (AMPAR). APP plays a role in NMDAR trafficking, enhancing its surface expression in the process^30,31^ while NMDAR activation decreases surface expression of APP, promoting amyloidogenesis^32^. In mice, overexpressing wild-type APP or APP with a Swedish double mutation, similar to cases of early-onset Alzheimer’s disease (EOAD) in humans, the expression and function of the AMPAR was reduced in neurons^33^. On the other hand, Gamma-aminobutyric acid (GABA) is the main inhibitory neurotransmitter in the brain operating through fast acting ionotropic GABA type A receptors (GABA_A_Rs) and slow metabotropic GABA type B receptors (GABA_B_Rs). GABA_A_Rs allow the bidirectional transport of Cl^−^ ions and GABA_A_R-mediated responses depend mainly on the cross-membrane Cl^−^ gradient which itself is determined by the intracellular Cl^−^ concentration ([Cl^−^]_i_). Cation-chloride transporters are considered to play a crucial role in Cl^−^ homeostasis. Under normal conditions the K^+^-Cl^−^ cotransporter KCC2 extrudes Cl^−^ from the cell while the Na^+^-K^+^-2Cl^−^ cotransporter NKCC1 promotes the intracellular accumulation of Cl^−^^34,35^. Imbalance in the expression or in the function of KCC2 or NKCC1 can lead to disrupted GABAergic inhibition. During neuronal maturation, expression of NKCC1 and KCC2 progressively decreases and increases, respectively. This change in expression induces a shift in GABAergic signaling from excitatory to inhibitory, also known as the GABA shift^36^. Not long ago, we demonstrated that the overexpression of APP in cortical cell cultures affects the GABA shift by decreasing KCC2 expression, without modifying NKCC1, resulting in a diminished inhibitory GABA response^37^. However, APP also seems to directly interact with KCC2, ensuring its stability and function at the cell surface^38^. GABA_B_Rs are composed of two subunits GABA_B1_ and GABA_B2_. The GABA_B1_ subunit is further divided in two isoforms GABA_B1a_ and GABA_B1b_ which differ in the presence of a tandem pair of extracellular domains, called sushi domain, located at the GABA_B1a_ isoform^39^. Interestingly, APP is highly expressed in GABAergic interneurons^40^ and was found to be part of the GABA_B_R complex^41^. Binding of APP to GABA_B_Rs promotes axonal trafficking, cell-surface expression of GABA_B_Rs, presynaptic inhibition, and at the same time protects APP from amyloidogenic cleavage by BACE1^42^. Compelling results published by Rice and colleagues revealed an additional function of APP concerning neurotransmission. Soluble APP, a product of both proteolytic cleavage pathways, was shown to bind to presynaptic GABA_B_Rs and regulate presynaptic neurotransmitter release^43^.

To investigate the role of APP in GABAergic signaling independent of Aβ plaques and APP mutations, we used a mouse model developed by Mucke and colleagues, that overexpresses wild-type human APP (hAPP_wt_; line I5) under the PDGF promoter, resulting in moderately (3–fourfold) increased levels of APP but with very low levels of Aβ peptides and no amyloid plaque formation^18,44^. In this study, we further characterized the hAPP_wt_ mouse model using behavioral experiments, with a focus on learning and memory as well as electrophysiology and report cognitive impairment, anxiety, and aberrant excitatory signaling in the hippocampus of hAPP_wt_ mice. We did not register any major changes in the expression of excitatory or inhibitory receptors or proteins involved in GABA production or efficacy. Further probing revealed changes in GABA content but not glutamate indicating diminished GABA function caused by APP overexpression.

## Results

### Characterization of APP metabolism in hAPP_wt_ mice

**Figure 1:**
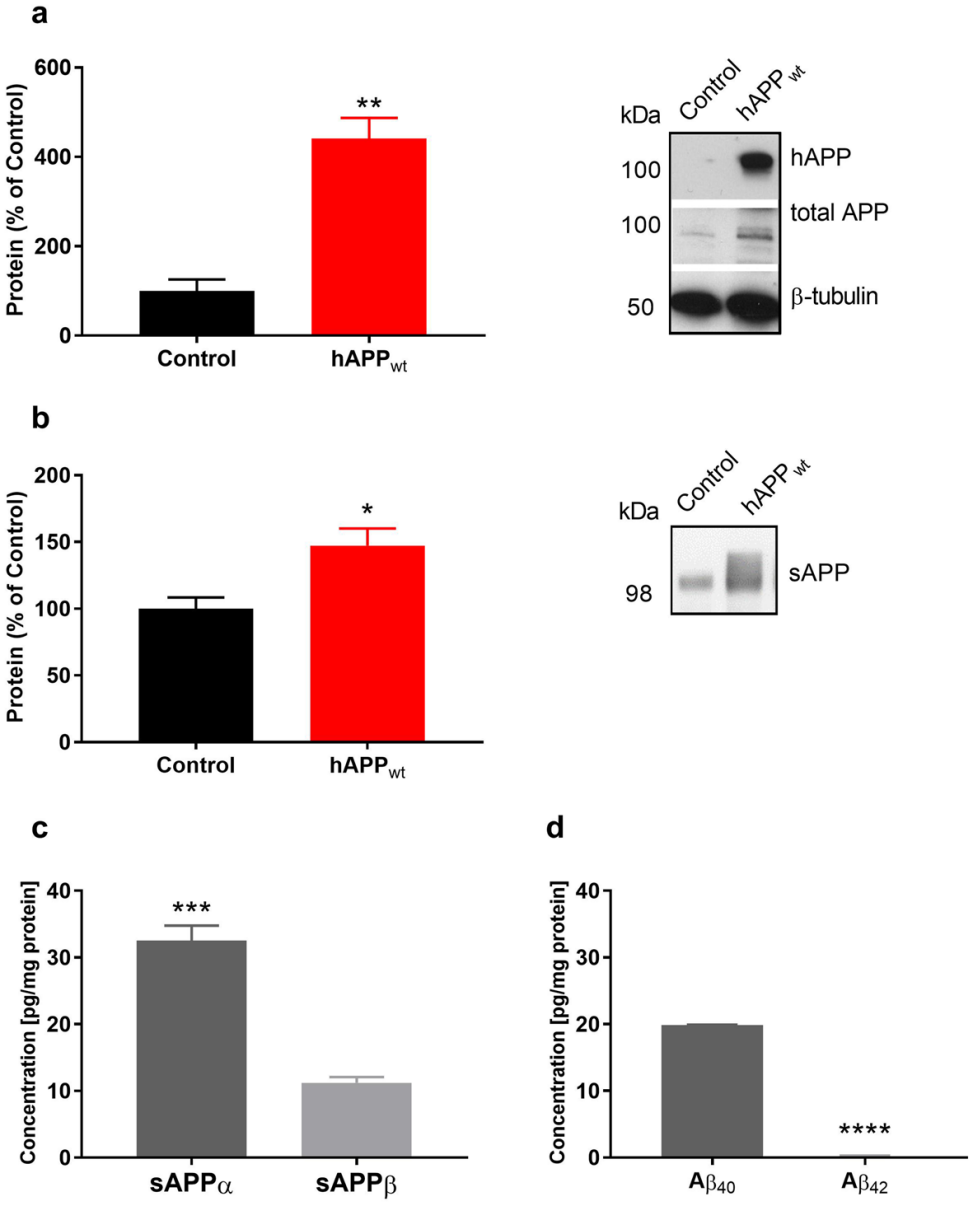
Characterization of APP metabolism in 6 month old male hAPP_wt_ mice and control littermates. (**a**) Quantification of total (mouse and human) APP (left panel) and representative western blots of human APP (hAPP) and total APP in hippocampal tissue lysates of 6 month old hAPP_wt_ (n = 4) and control (n = 4) animals (protein amount was normalized to β-tubulin and expressed as percentage). (**b**) Quantification and representative western blot of soluble mouse and human APP measured in medium collected from organotypic brain slice cultures of 6 months old hAPP_wt_ (n = 3) and control (n = 3) animals (protein amount was normalized to controls and expressed as percentage). (**c**) Quantitative determination of soluble human APP (sAPPα/sAPPβ) and (**d**) human amyloid peptides (Aβ_40_/Aβ_42_) in hippocampal tissue lysates of 6 month old male hAPP_wt_ (n = 4 for sAPPα/sAPPβ; n = 3 for Aβ_40_/Aβ_42_). No human sAPPα/sAPPβ or human Aβ_40_/Aβ_42_ was detected in control samples (ECLIA measurements were normalized to the protein content of each sample; Values are means ± SEM; *P ≤ 0.05, **P ≤ 0.01, ***P ≤ 0.001, ****P ≤ 0.0001, two-tailed unpaired t-test with Welch’s correction for **a/b**, two-tailed paired t-test for **c/d**).

We first confirmed the expression of human wild-type APP in 6 month old hAPP_wt_ with no detection in control samples (Fig. 1a). Using the APP C-terminus antibody recognizing both human and endogenous mouse APP (Sigma Aldrich), we measured the expression of total APP which was increased by four times in hAPP_wt_ mice compared to controls (P = 0.0018; Fig. 1a). Soluble endogenous mouse and human APP protein levels were determined using the anti-APP N-terminus clone 22C11 antibody (Merck). Analysis of media taken from organotypic brain slice cultures of 6 month old male hAPP_wt_ and control animals revealed a significant increase (P = 0.0447) in soluble mouse and human APP fragments in hAPP_wt_ animals compared to controls (Fig. 1b). Soluble APP fragments are generated in both APP cleavage pathways with sAPPα resulting from non-amyloidogenic cleavage and sAPPβ being the product of amyloidogenic processing. When comparing these two peptides, sAPPα is generally associated with beneficial functions and a possible therapeutic strategy for AD treatment, while sAPPβ is thought to have no positive functions at all^45^. Remarkably, both sAPP forms play a role in the regulation of presynaptic neurotransmitter release^43^. Since the discrimination of different sAPP species via western blot is rather difficult we set out to determine the human sAPP concentrations of both sAPPα and sAPPβ using an ECLIA assay (Mesoscale). Calculated levels of sAPPα/sAPPβ were normalized to protein amount of hippocampal samples taken from 6 month old male hAPP_wt_ mice and age-matched controls. We detected significantly more (P = 0.0009) sAPPα compared to sAPPβ with no detectable levels of human sAPP fragments in control samples (Fig. 1c). We additionally measured hippocampal levels of Aβ_40_ and Aβ_42_ at 6 months of age using an ECLIA assay (Mesoscale) specific for human Aβ peptides. hAPP_wt_ mice had almost non-detectable levels of Aβ_42_ (P ≤ 0.0001) and low amount of Aβ_40_, reaching 20 pg/mg protein while no human Aβ_40_/Aβ_42_ was detected in control samples (Fig. 1d). For comparison, we matched the recorded Aβ levels from hAPP_wt_ hippocampi to those observed in a well-established model of AD, the 5×FAD mice. In the latter, at the same age, Aβ_40_ levels reached around 600 pg/mg protein and 2500 pg/mg for Aβ_42_ on average (Suppl. Figure 1). We conclude that hAPP_wt_ mice exhibit increased levels of soluble APP but low levels of Aβ_40_/Aβ_42_.

### Mice overexpressing hAPP_wt_ exhibit cognitive impairment and anxious phenotype

**Figure 2:**
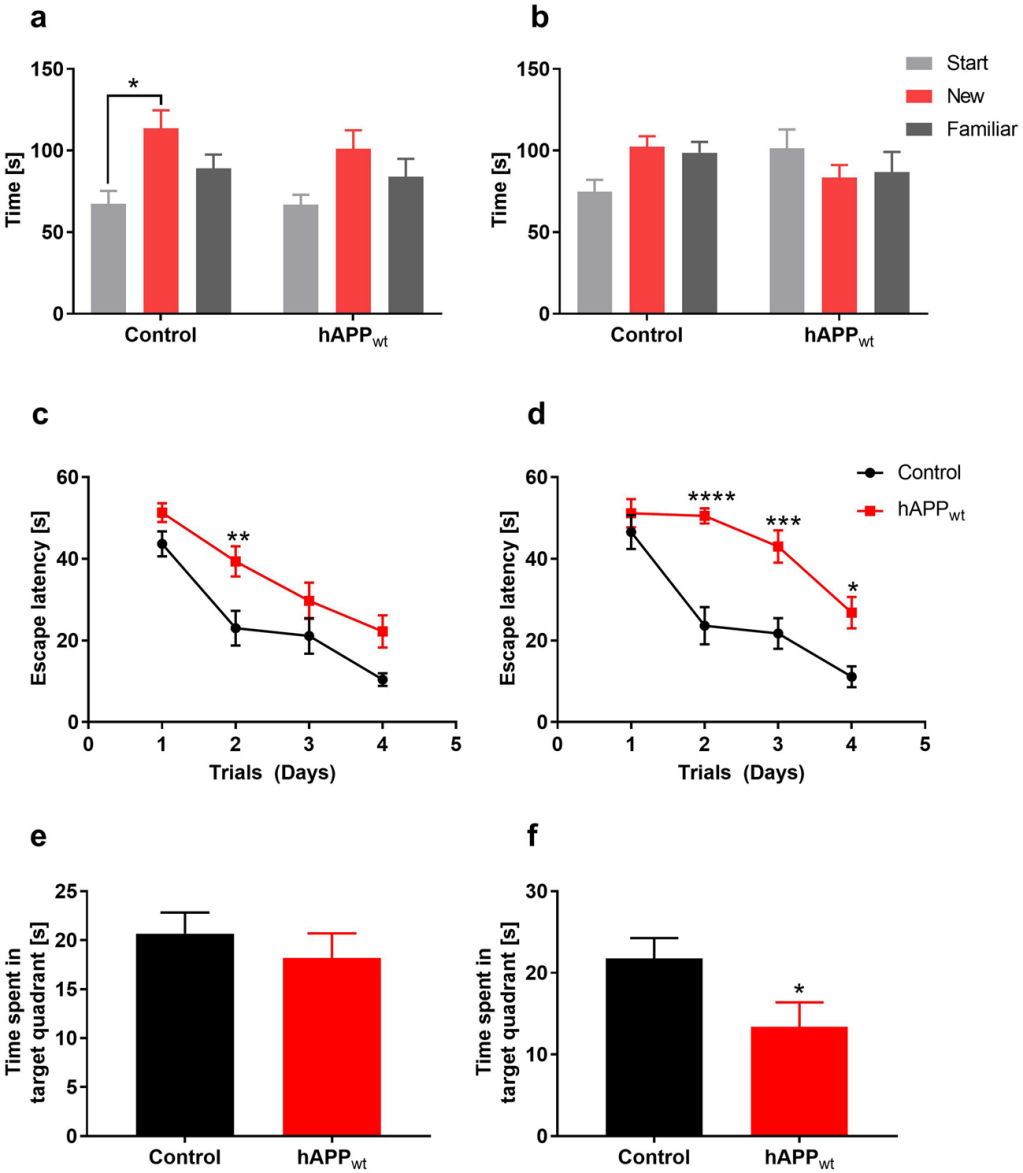
Performance of 6 month and 12 month old male hAPP_wt_ and control animals in behavioral tasks. (**a**) Recorded exploration time of the start, new and familiar arm of the modified Y-maze in 6 month old hAPP_wt_ (n = 16) vs. control (n = 12) mice and (**b**) in 12 month old hAPP_wt_ (n = 8) and control (n = 12) animals. (**c**) Recorded escape latency in the Morris water maze (MWM) compared between 6 month old hAPP_wt_ (n = 16) and control (n = 12) animals as well as (**d**) 12 month old hAPP_wt_ (n = 8) and control (n = 12) mice. (**e**) Recorded time spent in the target quadrant of the MWM during probe test (platform removed) of 6 month old hAPP_wt_ (n = 16) and control (n = 12) animals and (**f**) 12 month old hAPP_wt_ (n = 8) and control (n = 12) mice. (Values are means ± SEM; *P ≤ 0.05, **P ≤ 0.01, ***P ≤ 0.001, ****P ≤ 0.0001, two-way ANOVA with Sidak’s post-hoc test for **a–d**, two-tailed unpaired t-test for **e/f**).

**Figure 3:**
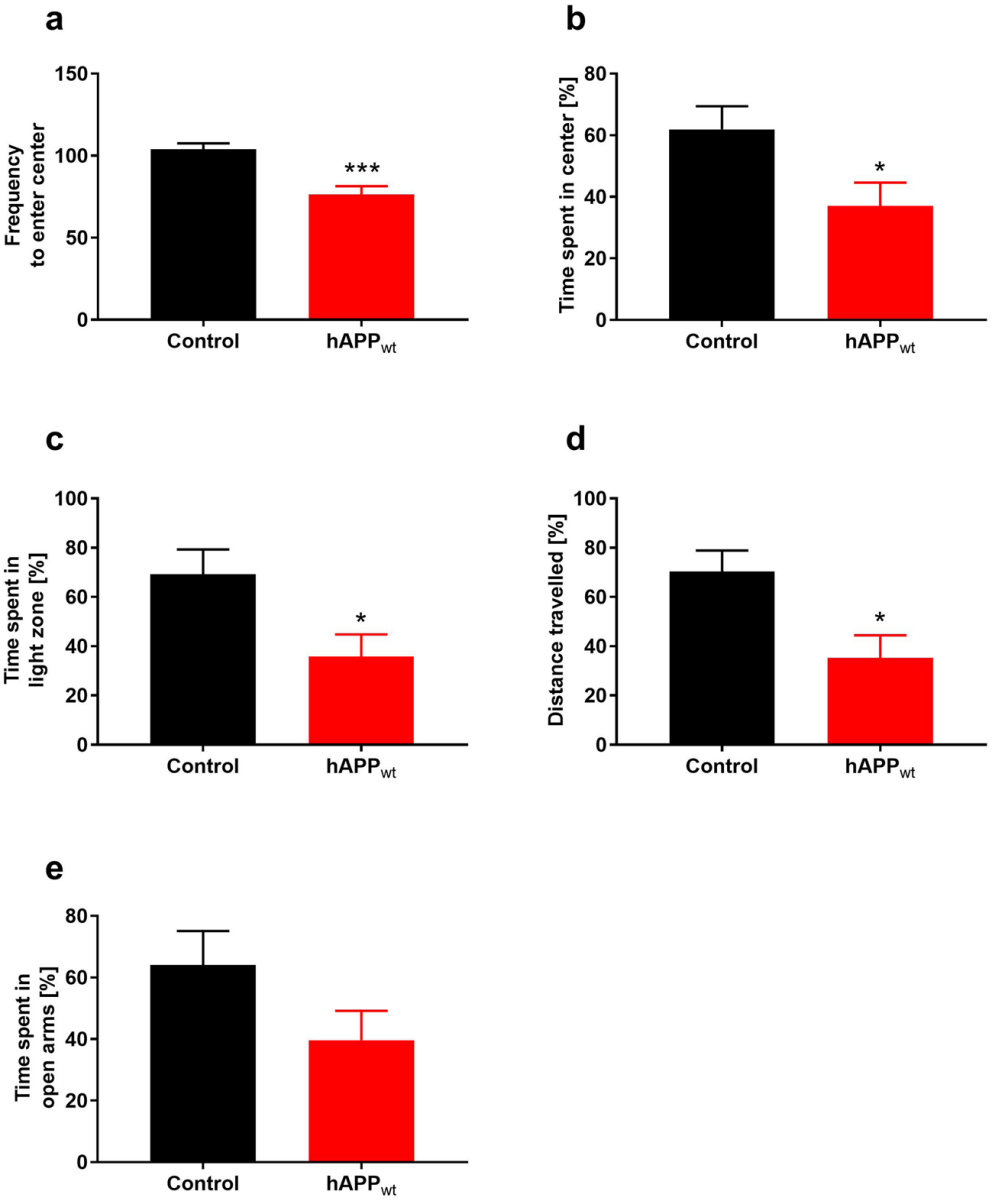
Assessment of anxiety-like behavior in 6 month old male hAPP_wt_ mice and corresponding controls. (**a**) Frequency to enter the center of the open field (OF) was compared between hAPP_wt_ (n = 16) and controls (n = 12). (**b**) Percentage of time spent in the center of the OF. (**c**) Percentage of time spent in the light zone of the light–dark test setup (LDT). (**d**) Distance travelled in the light zone of the LDT (n = 10 for hAPP_wt_ and n = 7 for controls). (**e**) Percentage of time spent in the open arms of the elevated plus maze (EPM) (n = 10 for hAPP_wt_ and n = 7 for controls). (Values are means ± SEM; *P ≤ 0.05, ***P ≤ 0.001, two-tailed unpaired t-test for **a** and two-tailed unpaired t-test with Welch’s correction for **b–e**).

Assessments of spatial reference memory and spatial working memory were performed using Morris water maze (MWM) and modified Y-maze. Open field (OF), light–dark test (LDT), and elevated plus maze (EPM) tests were used to determine exploration behavior, locomotor activity and unconditioned anxiety response. In the modified Y-maze, animals could explore the maze for 10 min while one of its three arms was blocked. Thirty minutes later, mice were introduced to the Y-maze, with all three arms open and accessible. For the modified Y-maze, 6 and 12 month old hAPP_wt_ and control littermates were used. At 6 months, control animals spent significantly more time (P = 0.0362) in the newly accessible arm compared to the start or familiar arm of the modified Y-maze, while hAPP_wt_ mice did not show any significant preference for any arm, pointing to impaired spatial working memory (Fig. 2a). At 12 months of age, control animals still showed a tendency, although not significant, to prefer the newly exposed arm compared to the start and familiar arm, while hAPP_wt_ mice did not show any significant preference for any arm of the Y-maze (Fig. 2b). In the MWM, mice learn the location of a hidden platform using visual cues while the escape latency (time to reach the platform) is measured. On the first trial day, all hAPP_wt_ and control mice, reached the platform in about 50 s with no visible differences in swimming speed, suggesting the absence of any major locomotor defect. At 6 months of age, hAPP_wt_ mice exhibited a significantly longer escape latency (P = 0.009) on day 2 of the MWM test when the platform was invisible, but overall, still learned normally (Fig. 2c). The probe test that was performed on day 5 was normal, suggesting a normal spatial reference memory (P = 0.4756; Fig. 2e). At 12 months of age however, the escape latency of hAPP_wt_ mice was significantly longer on day 2 (P ≤ 0.0001), 3 (P = 0.0009), and 4 (P = 0.0215) of the MWM (Fig. 2d). Additionally, the probe test showed a significant decrease (P = 0.0480) in time spent in the target quadrant when the platform was completely removed from the MWM (Fig. 2f) suggesting an impairment in learning ability and an alteration in reference memory. Willingness to explore new environments, locomotor activity as well as anxiety was measured in the OF experiment and revealed that 6 month old hAPP_wt_ mice entered the center of the OF significantly less frequently (P = 0.0002; Fig. 3a) and spent significantly less time (P = 0.0299) in the center of the OF compared to control littermates (Fig. 3b). These mice had however a similar locomotor activity (9894 ± 265 cm / 20 min for control mice n = 12 vs 9235 ± 426 cm / 20 min for hAPP_wt_ mice, n = 16). Measurement of anxiety-like behavior was performed using the LDT and the EPM based on natural aversion of mice to brightly illuminated areas, and mild stressors such as novel environment. At 6 months of age, hAPP_wt_ mice spent significantly (P = 0.0261 time in light area; P = 0.0142 distance moved) less time in the illuminated area compared to controls (Fig. 3c/d). In the EPM, 6 month old hAPP_wt_ mice tended to spend less time in the open arms but the difference did not reach statistical significance (P = 0.1196; Fig. 3e). Overall, hAPP_wt_ mice exhibited impaired spatial reference learning and memory that started at 6 months of age and fully manifested at 12 months, indicating impaired hippocampal function. Compared to controls, 6 month old hAPP_wt_ animals also exhibited some reluctance to enter the center of the OF and avoided the illuminated area of the LDT suggesting increased anxious phenotype.

### Overexpression of hAPP_wt_ leads to hippocampal neuronal death

**Figure 4:**
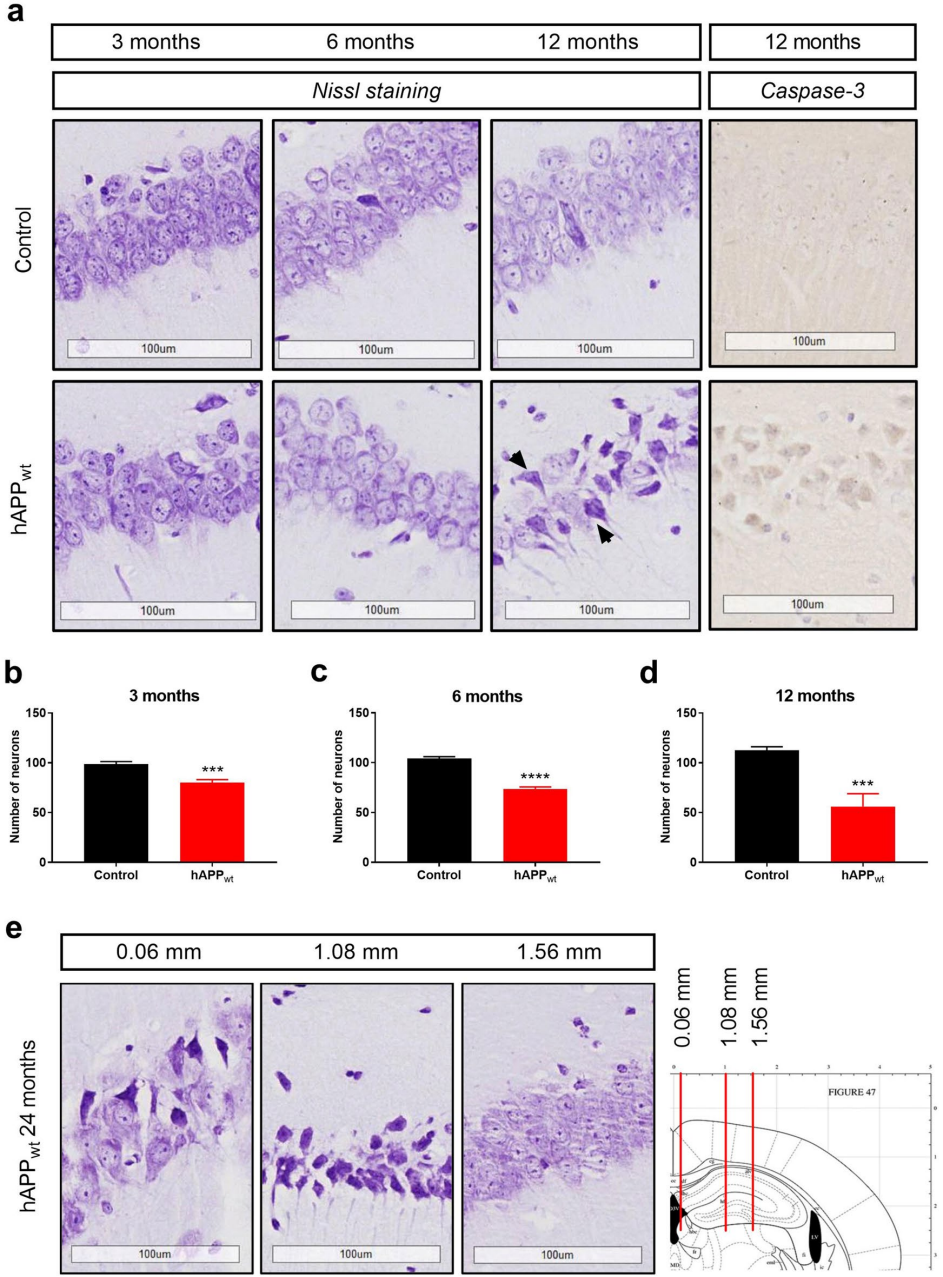
Morphological changes in the CA1 hippocampal region in male hAPP_wt_ and age-matched control animals at 3–24 months of age. (**a**) Comparison of Nissl stained sagittal brain sections of the hippocampal CA1 area between hAPP_wt_ and age-matched control littermates, starting at 3 months and progressing to 12 months of age. Arrows mark the presence of dark Nissl stained neurons in hAPP_wt_ mice at 12 months of age, which is a sign of neuronal death. Neuronal degeneration and death were confirmed by caspase-3 staining in 12 month old hAPP_wt_ animals. (**b–d**) Quantification of surviving neurons in the CA1 area of 3, 6, and 12 month old hAPP_wt_ animals and age matched littermates. Neurons with round and palely stained nuclei were considered as viable, while shrunken and dark stained cells were considered as dead. Data are expressed as the number of surviving neurons per analyzed CA1  section (300 μm × 300 μm) in each age group for hAPP_wt_ (n = 8) and control (n = 8) mice. (**e**) Nissl stained sagittal brain sections of the hippocampal CA1 area in 24 month old hAPP_wt_ mice reveal a lateral progression of neuronal degeneration, which is evident by the presence of dark Nissl stained neurons. Sections were taken later at 0.06 mm, 1.08 mm, and 1.56 mm; values are means ± SEM; ***P ≤ 0.001, ****P ≤ 0.0001, two-tailed unpaired t-test).

The hippocampus plays a key role in memory consolidation and is one of the primary structures for functional and pathological alterations in AD. We therefore analyzed possible changes in hippocampal morphology using hAPP_wt_ mice and age-matched controls. Nissl stained sagittal brain  sections (5 μm) were obtained at 3, 6, 12 and 24 months of age. Compared to age-matched control littermates, the hippocampus of hAPP_wt_ mice appeared to be thinner with missing or dead neurons specifically in the CA1 hippocampal area (Fig. 4a). The quantification of the number of viable neurons in the hippocampal CA1 region in hAPP_wt_ and control littermates at 3, 6, and 12 months of age is presented on Figs. 4b-d. In all age groups, hAPP_wt_ mice presented significantly less neurons (3 months P = 0.0004; 6 months P ≤ 0.0001; 12 months P = 0.0009) in the CA1 area compared to controls suggesting neuronal loss. Furthermore, at 12 months of age, the Nissl staining revealed dark shrunken neurons in hAPP_wt_ brain slices which typically indicate neuronal death (Fig. 4a)^46^. The presence of dead neurons was confirmed in 12 month old hAPP_wt_ mice by positive caspase-3 staining, a cysteine-aspartic acid protease typically involved in cell death. Additionally, hippocampal sections were taken from 24 month old hAPP_wt_ animals at different lateral markers to investigate further neuronal loss (Fig. 4e). The Nissl stained hippocampal sections revealed the same dark shrunken neurons, that seemed to further advance into the hippocampus along the lateral axis indicating a spread of neurodegeneration at 24 months of age. Taken together, the morphological changes in hAPP_wt_ mice could partially explain the cognitive impairment observed in hAPP_wt_ mice.

### Overexpression of hAPP_wt_ leads to increased excitability and spontaneous spiking activity

**Figure 5:**
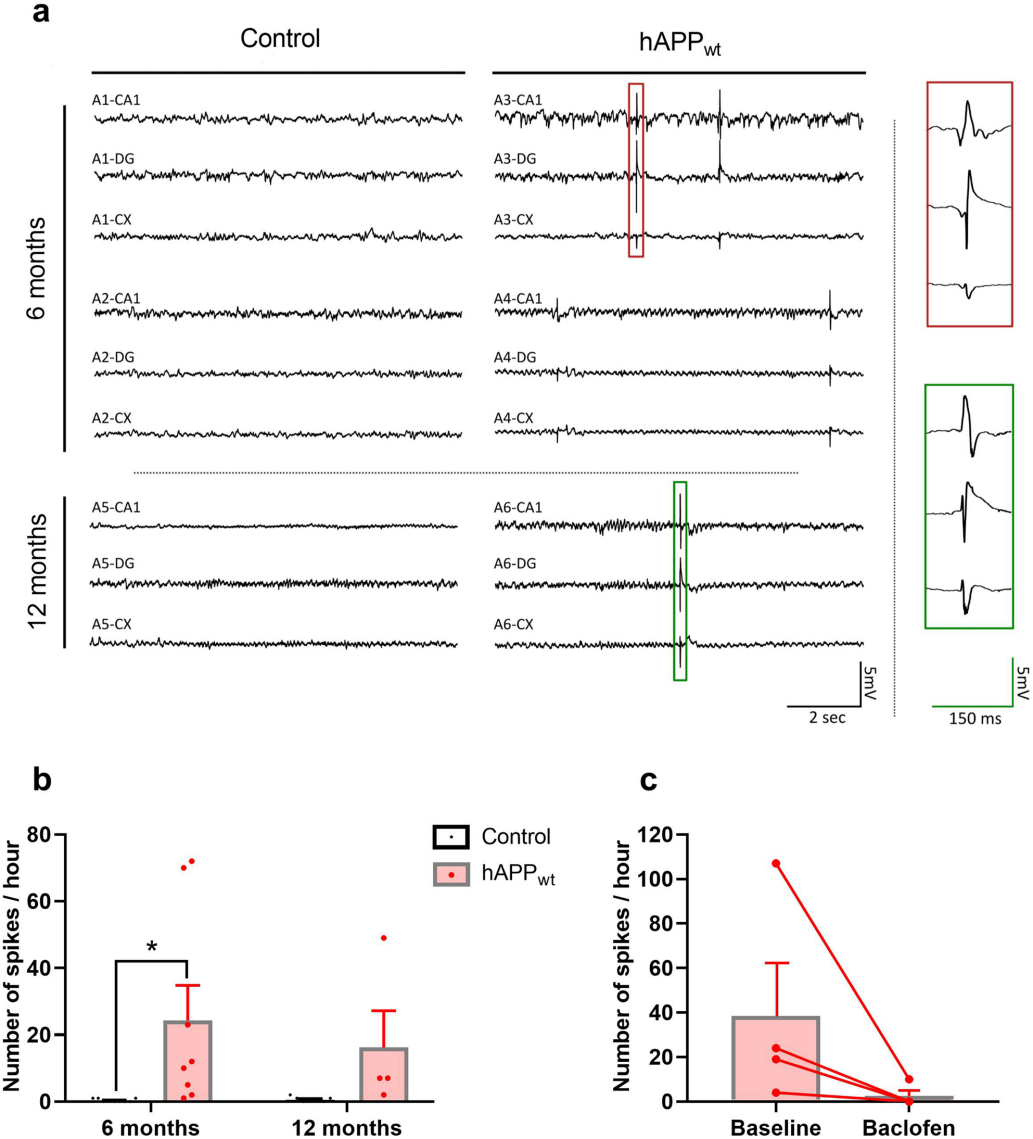
EEG recordings in 6 and 12 month old male hAPP_wt_ and control mice. (**a**) Electroencephalography (EEG) recordings of spontaneous spike activity documented in the hippocampus (CA1 area), dentate gyrus (DG), and cortex (CX) in 6 month old hAPP_wt_ (traces A3, A4) (n = 8) and controls (traces A1, A2) (n = 9) as well as 12 month old hAPP_wt_ (trace A6) (n = 4) and control (trace A5) (n = 7) mice. (**b**) Quantification of annotated number of spikes per hour in 6 and 12 month old hAPP_wt_ and control mice with individual values for each animal represented by dots. (**c**) Abolishment of spiking activity after baclofen treatment in 6 month old hAPP_wt_ (n = 4) mice. (Values are means ± SEM; *P ≤ 0.05, two-way ANOVA with Sidak’s post-hoc test).

**Figure 6:**
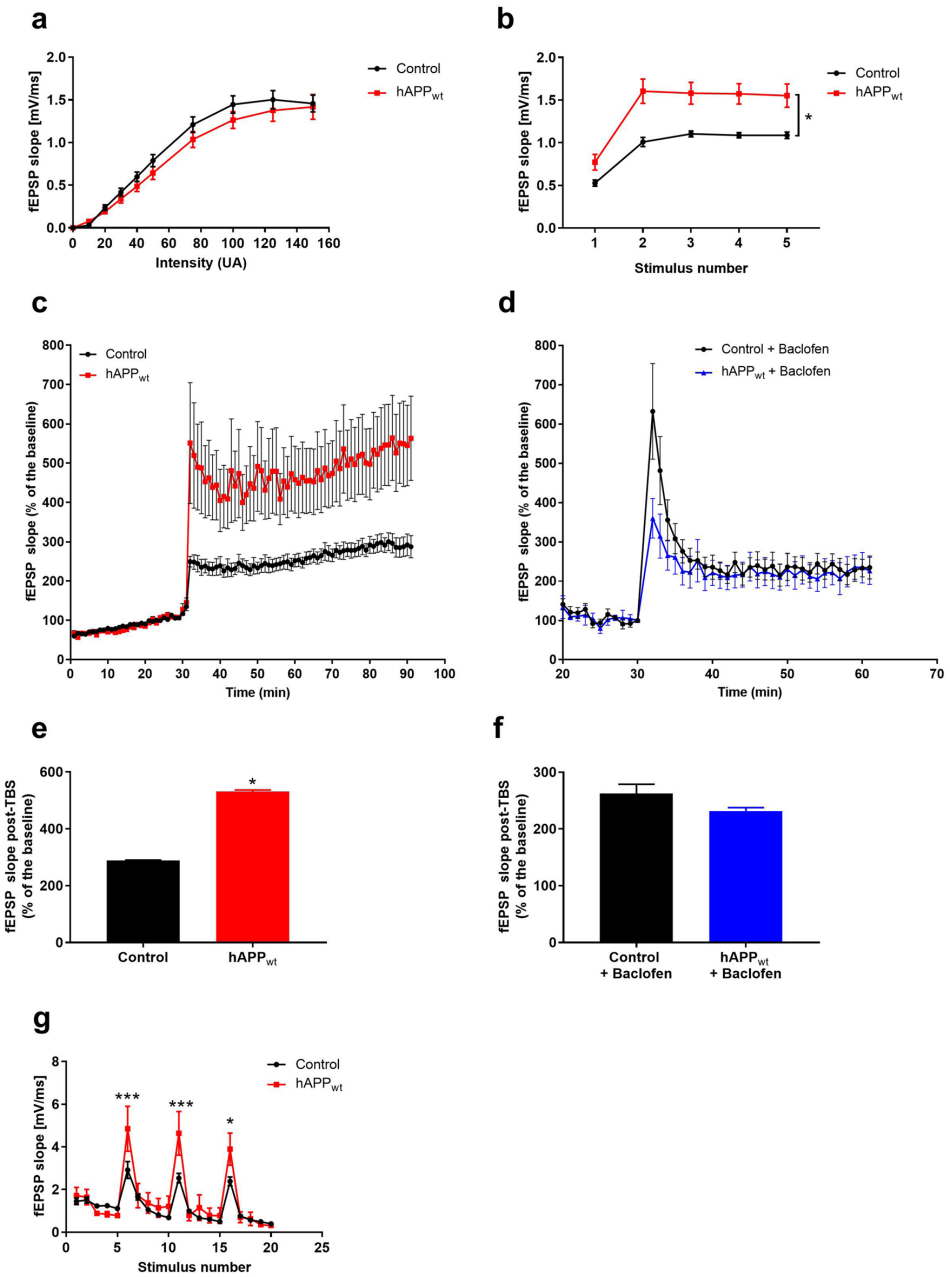
Increased LTP and short-term facilitation in 6 month old male hAPP_wt_ animals. (**a**) Current curves of hAPP_wt_ (n = 51) and controls (n = 48). (**b**) Short-term facilitation at 20 Hz stimulation frequency in hAPP_wt_ (n = 8) brain slices vs. controls (n = 5). (**c**) LTP in hippocampal SC-CA1 pathway electrically stimulated by TBS in hAPP_wt_ (n = 14) compared to controls (n = 12). (**d**) Rescue of increased LTP response in hAPP_wt_ mice (n = 8) compared to controls (n = 7) after perfusion with 100 μM baclofen. (**e**) Quantification of LTP in hippocampal slices of hAPP_wt_ and controls after TBS introduction (corresponding to **c**) and (**f**) Quantification of LTP after baclofen perfusion (corresponding to **d**). (**g**) Increased fEPSP responses to TBS in hAPP_wt_ (n = 5) animals compared to controls (n = 4). (Values are means ± SEM; *P ≤ 0.05, ***P ≤ 0.001, two-way ANOVA with Sidak’s post-hoc test for **b–g**, two-tailed unpaired t-test for **a**).

Cognitive impairment and memory problems are commonly connected to impaired neuronal plasticity or aberrant excitability^47,48^. This was investigated in vivo and ex vivo, on brain slices. Intracranial EEG was monitored using electrodes implanted in CA1 hippocampal area (stratum pyramidale), dentate gyrus (DG; granule cell layer) and cortex of hAPP_wt_ and control animals at 6 and 12 months of age. EEG recordings revealed abnormal spikes in hAPP_wt_ at 6 and 12 months but not in controls (P = 0.0206; Fig. 5 a/b). This epileptiform activity largely decreased after treatment with baclofen, a pan-GABA_B_R agonist (Fig. 5c). Recorded spikes were unaccompanied by abnormal movements or convulsive seizures. To further characterize a possible impairment in neuronal excitability or plasticity, we performed extracellular field potential recordings on hippocampal slices of hAPP_wt_ and control animals at 6 months of age. Schaffer collaterals (SCs) were stimulated electrically, and field excitatory postsynaptic potentials (fEPSPs) were recorded in the stratum radiatum of CA1 hippocampal region (Fig. 6). The relationship between the stimulus intensity and the fEPSP slope was similar in slices from both genotypes (P = 0.7417; Fig. 6a). To study whether hAPP_wt_ overexpression affects presynaptic properties of CA3-CA1 synapses, we performed short-term facilitation induced by bursts of five stimuli at a frequency of 20 Hz (Fig. 6b). The observed short-term facilitation was significantly (P = 0.0120) larger in brain slices from hAPP_wt_ mice compared to controls, suggesting a decreased probability of neurotransmitter release at synapses. To induce long-term potentiation (LTP), we used a theta-burst stimulation (TBS) consisting of four trains of five pulses (at 100 Hz) separated by an interburst interval of 200 ms. In slices from control animals, TBS induced an increase of fEPSP response size to about 250% of the initial response (Fig. 6c). This potentiated response persisted up to the end of the experiment (at least one hour). In hAPP_wt_ mice, LTP was significantly increased by a factor of about 2 in comparison to control mice (P = 0.0362; Fig. 6c/e). Treatment of hAPP_wt_ brain slices with baclofen (100 μM) diminished this abnormally elevated LTP observed in hAPP_wt_ (Fig. 6d/f). The response to TBS itself was in fact modified: in hAPP_wt_ brain slices, the responses to the second (P = 0.0006), third (P = 0.0001) and fourth (P = 0.0196) bursts of stimulation were globally increased in comparison to the responses to the first burst (Fig. 6g). This was not observed in control hippocampal slices.

### Overexpression of hAPP_wt_ does not alter the expression of inhibitory or excitatory receptors

**Figure 7:**
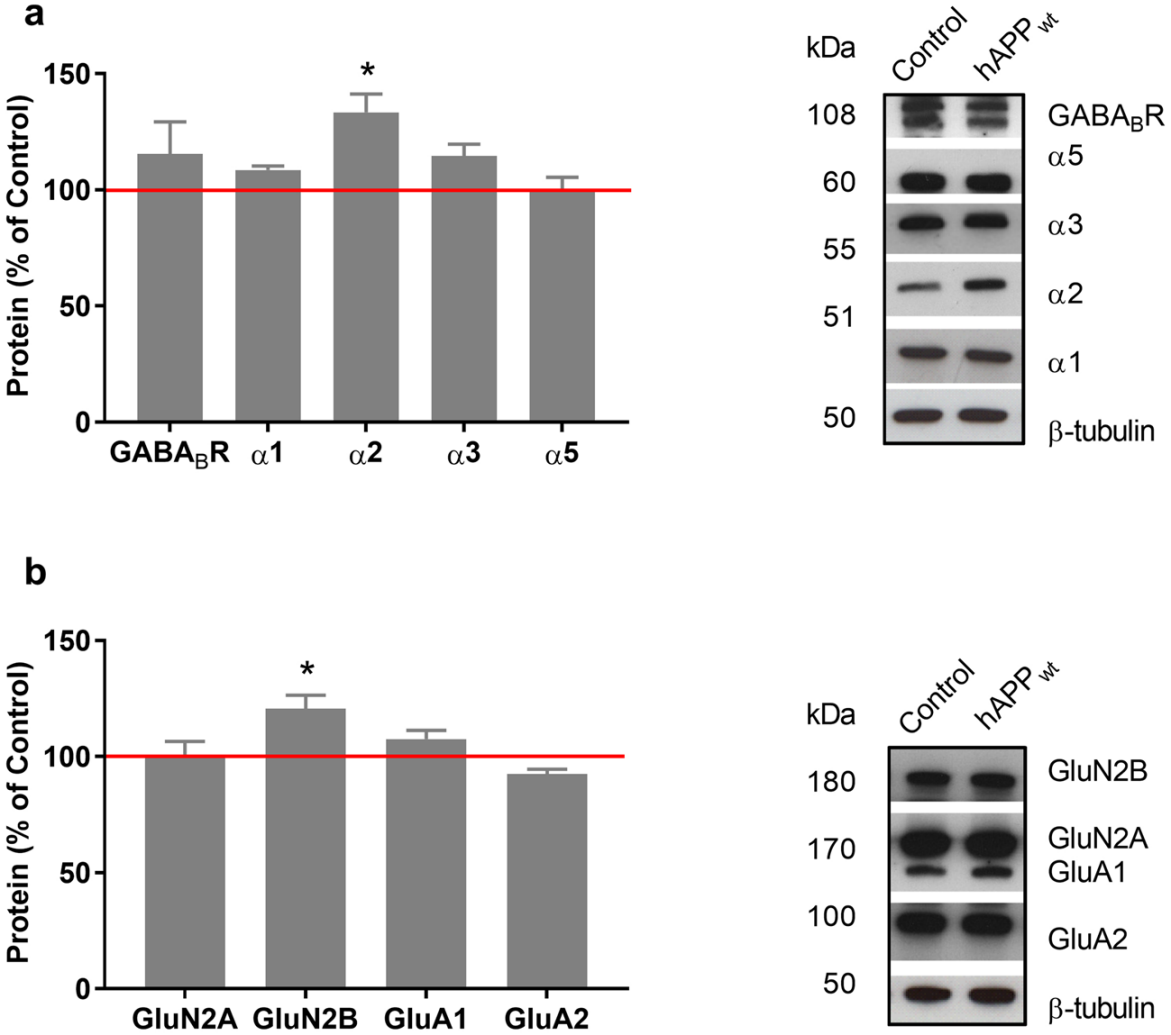
Protein levels of the main GABAergic and glutamatergic receptors in 6 month old male hAPP_wt_ mice. (**a**) Quantification and representative western blots of GABAergic receptor protein levels, including GABA_B_R and GABA_A_R α1, α2, α3 and α5 subunits, measured in hippocampal tissue lysates of hAPP_wt_ (n = 4) and control (n = 4) animals. (**b**) Quantification and representative western blots of glutamatergic receptor subunits of NMDA receptor (GluN2A, GluN2B) and AMPA receptor (GluA1, GluA2) measured in hippocampal tissue lysates of hAPP_wt_ (n = 4) and control (n = 4) animals. Protein amount was normalized to β-tubulin and expressed as percentage (Values are means ± SEM; *P ≤ 0.05, two-tailed unpaired t-test with Welch’s correction).

We analyzed possible alterations caused by hAPP_wt_ overexpression in proteins directly implicated in the process of inhibitory and excitatory signaling. GABA mediates its actions via fast acting ionotropic GABA_A_Rs and slow metabotropic GABA_B_Rs. GABA_A_Rs exhibit a pentameric channel structure consisting of five subunits arranged around a central pore. The subunits can be subdivided into seven structurally related subfamilies α 1–6, β 1–4, γ 1–3, ε, δ, π, ρ 1–3 and θ^49^. Since the composition of GABA_A_Rs is very variable, we focused on the most abundant subunits in the hippocampus. GABA_A_R α-subunits are amply distributed in the hippocampus with α1, α2, α3 and α5 being the most abundant^50^. In contrast, slow metabotropic GABA_B_Rs exert their regulatory effects on synaptic transmission by acting as autoreceptors and heteroreceptors by inhibiting neurotransmitter release at GABAergic and glutamatergic synapses, respectively. We investigated hippocampal protein levels of GABA_A_R α1, α2, α3 and α5 as well as GABA_B_R using western blot. No significant changes were detected in respect to α1, α3 and α5 subunits apart from a slight increase in α2 (P = 0.0191; Fig. 7a). Separately, NMDARs are essential contributors to glutamatergic excitatory synaptic transmission, as well as to several forms of synaptic plasticity in the adult brain. Activation of NMDARs is also essential for the recruitment of AMPARs into silent synapses and for other types of Ca^2+^-dependent synaptic plasticity^51,52^. Using western blot, we analyzed the possible effect of APP overexpression on glutamatergic receptors, focusing on most abundant subunits of NMDAR (GluN2A and GluN2B) and AMPAR (GluA1 and GluA2) based on their expression in the CNS^53^. At 6 months of age hippocampal protein levels of AMPAR subunits GluA1 and GluA2 remained unchanged in hAPP_wt_ mice as well as controls (Fig. 7b). As for NMDAR subunits we only detected a small increase in GluN2B (P = 0.0456) while GluN2A remained unchanged (Fig. 7b). Taken together, we did not detect any major changes in GABAergic/glutamatergic receptor subunits except a slight increase in GABA_A_R α2 subunit and in NMDAR subunit GluN2B.

### Overexpression of hAPP_wt_ decreases GABA but not glutamate amount

**Figure 8:**
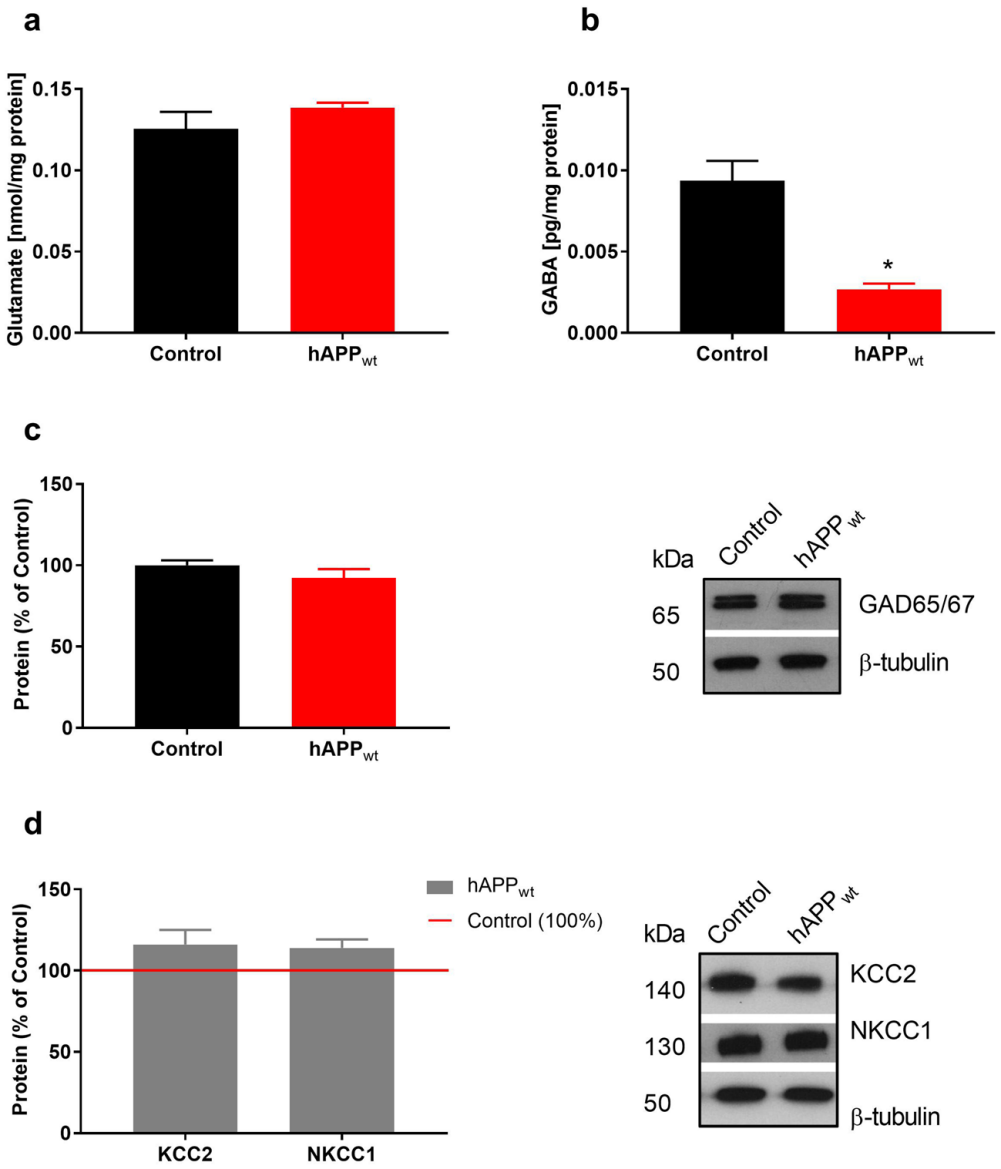
Quantitative determination of GABA content, production, and efficacy in 6 month old male hAPP_wt_ mice. (**a**) ELISA measurements of free glutamate content in hippocampal tissue lysates of hAPP_wt_ (n = 4) and control (n = 4) animals. (**b**) GABA ELISA measurements in hippocampal tissue lysates of hAPP_wt_ (n = 4) and control (n = 4) mice. (**c**) Quantification and representative western blots of two glutamic acid decarboxylase isoforms (GAD65 and GAD67) using a combined antibody on hippocampal tissue lysates of hAPP_wt_ (n = 3) and controls (n = 3). (**d**) Protein levels of KCC2 and NKCC1 were obtained from hippocampal tissue lysates hAPP_wt_ (n = 4) and control (n = 4) mice. Quantification and representative western blots are displayed. For all western blots, the protein amount was normalized to β-tubulin and expressed as percentage. ELISA measurements were normalized to protein content of each sample (Values are means ± SEM, *P ≤ 0.05, two-tailed unpaired t-test with Welch’s correction).

Hippocampal contents of GABA and glutamate were measured using specific ELISA assays. Glutamate content in hAPP_wt_ hippocampi did not differ from control samples (Fig. 8a). In contrast, hippocampal GABA content was significantly (P = 0.0237) decreased in mice overexpressing hAPP_wt_ (Fig. 8b). These observations lead us to hypothesize that a decrease of GABA release might cause overexcitability and the increased response to TBS.

### Overexpression of hAPP_wt_ does not alter the amount of GAD65/67

The detected decrease in GABA could also arise from impaired GABA production. GABA is synthesized from glutamate by the enzyme glutamic acid decarboxylase (GAD65 and GAD67), in particular by GAD65, which is preferentially localized at GABAergic nerve terminals. We assessed hippocampal protein levels of GAD65/67 via western blot in 6 month old hAPP_wt_ and control mice (Fig. 8c). No significant changes were detected concerning the protein amount of GAD65/67, suggesting that GABA production capacity is not impaired in hAPP_wt_ animals.

### Overexpression of hAPP_wt_ does not influence GABA shift in vivo

Regulation of cognitive processes depends on the fine tuning between excitatory and inhibitory neurotransmission. GABAergic neurotransmission is determined by the electrochemical gradient that depends on the intracellular chloride concentration [Cl^−^]_i_. The main players in regulating the [Cl^−^]_i_ in neurons are KCC2, the primary Cl^−^ extruder and NKCC1, the predominant Cl^−^ importer. To assess the possible influence of hAPP_wt_ overexpression on GABA efficacy, we measured hippocampal protein levels of KCC2 and NKCC1 in hAPP_wt_ mice and controls at 6 months of age (Fig. 8d). No significant changes in either KCC2 or NKCC1 were detected in the hippocampi of hAPP_wt_ animals compared to controls, leading to the conclusion that mechanisms controlling the Cl^−^ concentration are not impaired and therefore the ability of GABA to inhibit neuronal excitability should not be affected by the overexpression of hAPP_wt_.

### Rescue of increased excitability in hAPP_wt_ mice with CGP36216

**Figure 9:**
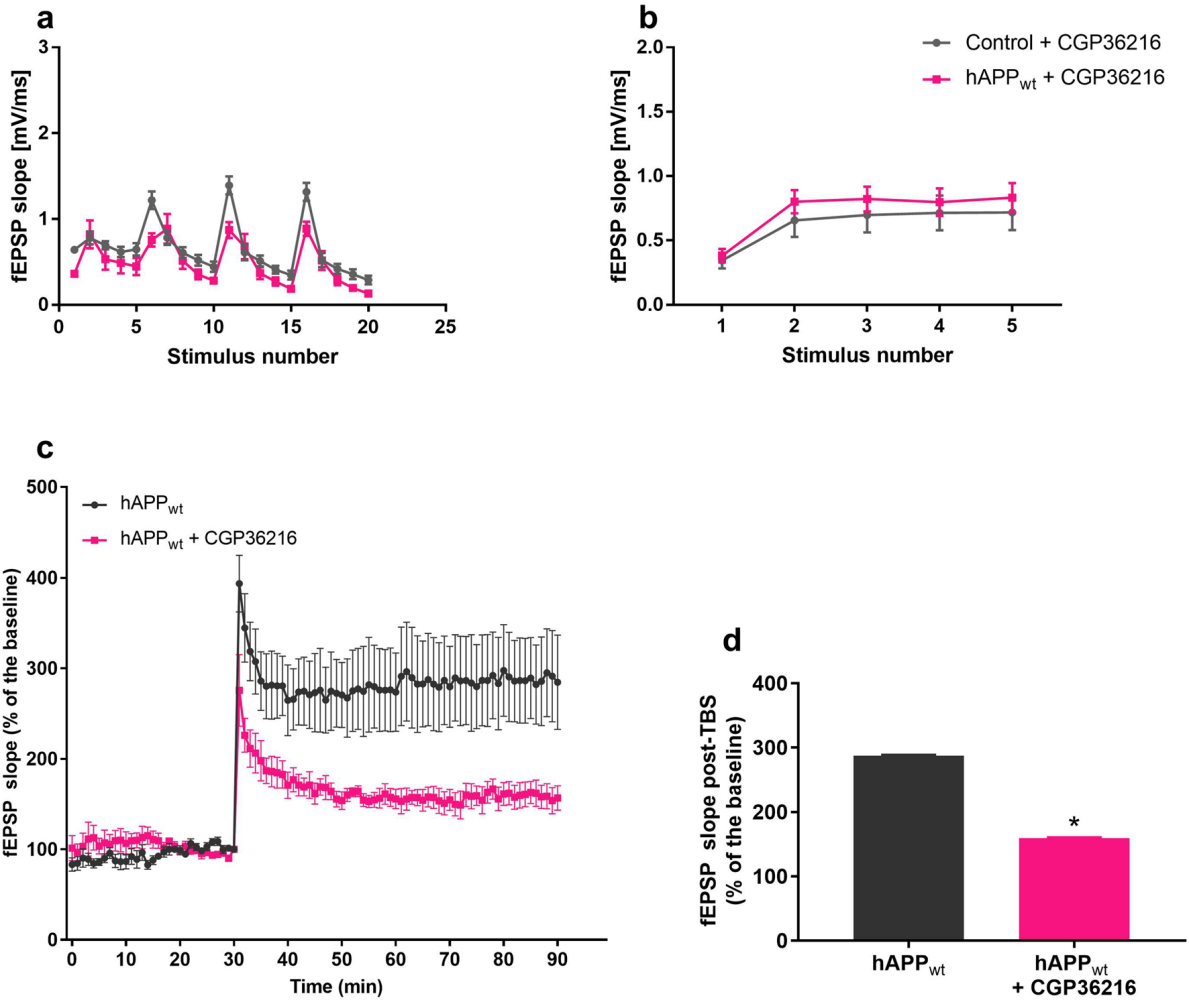
Rescue of increased excitability in 6 month old hAPP_wt_ mice with CGP36216. (**a**) Rescue of elevated TBS response in hippocampal slices of hAPP_wt_ (n = 5) vs. control (n = 5) and (**b**) Recovery of increased response to short-term facilitation during perfusion with 500 μM CGP36216 in hippocampal slices of hAPP_wt_ (n = 6) and control (n = 6) animals. (**c**) Rescue of increased LTP recorded in hAPP_wt_ mice (n = 5) and hAPP_wt_ mice (n = 5) perfused with 500 μM CGP36216. (**d**) Quantification of LTP with CGP36216 perfusion (corresponding to **c**). (Values are means ± SEM; *P ≤ 0.05, two-way ANOVA with Sidak’s post hoc test).

As mentioned before, we observed increased response to TBS in hippocampal slices of hAPP_wt_ mice at 6 months of age. This effect has been related to the activation of presynaptic GABA_B_ autoreceptors that inhibit GABA release and disinhibit the response. Increased response to TBS was completely rescued by application of 500 μM CGP36216, a specific antagonist of GABA_B_R that acts only on presynaptic GABA_B_Rs^54^ (Fig. 9a). Similarly, the exaggerated short-term facilitation observed in hAPP_wt_ hippocampal slices in response to five stimuli at 20 Hz returned to normal level after application of 500 μM CGP36216 (Fig. 9b). Moreover, TBS-induced LTP that was shown to be exaggerated in 6 month old hAPP_wt_ mice, was largely reduced by the treatment with CGP36216 (P = 0.0329; Fig. 9c/d).

## Discussion

It is widely established that APP mutations, altering Aβ production, can cause EOAD but the involvement of APP per se in its progression is not well understood^55^. However, synaptic function is affected at earliest stages of AD, partially independent from Aβ plaque formation and accompanied by disruption of excitatory/inhibitory balance in animal models as well as patients^6,7,48,56^. We were interested to see if and how APP manages to modulate excitatory and/or inhibitory neurotransmission in the absence of APP mutations or Aβ plaque formation. In this study we used a mouse model that overexpresses moderate levels of wild-type human APP (fourfold compared to wild-type animals) but generates very low levels of Aβ_40_/Aβ_42_ with no evident plaque formation^18,44,57^. The APP overexpression profile is comparable to a rare form of EOAD, in which the *APP* gene is duplicated (*Dup-APP*). In *Dup-APP*, chromosome 21 duplications can vary in size with the smallest known duplication containing an additional copy of *APP* without any other gene duplication^58,59^. Although the increase of APP is relatively small compared to our mouse model, individuals with *Dup-APP* still experience seizures^60^. In DS patients, APP overexpression occurs due to triplication of chromosome 21. Interestingly, both neurodegenerative diseases share a common profile and are both associated with seizures and epilepsy^61^. In our study, morphological examination of hAPP_wt_ and control mice brains, revealed a decrease of pyramidal cells in the CA1 hippocampal region with distinct cell death evidenced by caspase-positive cells in hAPP_wt_ mice at 12 months of age. Neuronal loss in this particular model has been reported before^57^, but it is still not clear if GABAergic and glutamatergic neurons are affected equally. Since we did not observe any major changes in the expression of GABAergic/glutamatergic receptors or in GABA production enzymes, it is highly likely that both neuronal populations are affected. Impairment in adult neurogenesis due to overexpression of hAPP_wt_ could also be partially responsible for the neuronal decline in the hippocampi of 3, 6, and 12 month old hAPP_wt_ animals^62^. The decrease in newborn neurons was more acute in hAPP_wt_ (the line I5 we used in the present paper) compared to a similar model of APP overexpression, the hAPP-J20 mouse line, that overexpresses APP with a Swedish-Indiana mutation and exhibits high Aβ levels with amyloid plaque formation, therefore suggesting that hAPP itself more than Aβ is an important factor inhibiting adult neurogenesis^44,62^. Additionally, moderate levels of hAPP_wt_ were sufficient to induce electrophysiological abnormalities. EEG recordings indeed revealed abnormal synchronous spiking activity in the DG, CA1 area, and cortex of 6 and 12 month old hAPP_wt_ animals, while no such activity was observed in controls. These changes occurred at very low Aβ levels and in the absence of amyloid plaques, suggesting the importance of hAPP and its non-amyloid metabolites. However, it has been reported that even picomolar concentrations of Aβ can lead to increased excitability^63,64^. In a recent study, Johnson et al. showed that familial AD-linked APP mutations could cause non-convulsive epileptiform activity in the absence of APP overexpression but that overexpression of hAPP_wt_ could cause similar dysfunctions in the absence of such mutations^18^. This reproduced the situation observed in patients presenting APP mutations (some forms of AD) or APP overexpression (e.g. gene duplication) and showed that hAPP_wt_ itself and its metabolites contribute to EEG abnormalities and to the hyperexcitability state, as it was suggested previously^17,18^. APP cleavage by ADAM10 or BACE1 liberates soluble APP fragments such as sAPPα/sAPPβ as well as membrane bound carboxyl-terminal fragments CTFα/CTFβ that serve as precursors to APP intracellular domain fragments (AICDs). Simón and colleagues detected increased levels of CTFs in hAPP_wt_ mice as well and further established that significantly more CTFα is produced compared to CTFβ, leading to the conclusion that APP is largely processed in the non-amyloidogenic pathway^57^. Besides increased CTFs, hAPP_wt_ exhibited elevated levels of APP intracellular domains (AICDs)^57^ which can be a product of both APP processing pathways and can cause alterations in hippocampal network oscillations and cognitive impairment^65^. Furthermore, hAPP_wt_ mice show increased levels of phosphorylated tau^57^ which could be the result of APP overexpression or increased levels of APP metabolites that cannot be excluded as a possible cause for neurodegeneration or increased excitability observed in hAPP_wt_ animals^66^. APP is present at synaptic sites which makes it a highly probable binding partner for receptors and gives it the capability to affect neurotransmission. Anatomical studies have shown that GABA_A_R α-subunits are highly expressed in the hippocampus with large population of GABA_A_Rs containing α1, α2, α3, and α5^67,68^. In AD patients, GABAergic signaling is altered with a decreased profile of various GABA_A_R subunits^69^. In mice lacking APP, GABA_A_R α1 is significantly decreased as well^38,70^. However, in hAPP_wt_ mice, α1 protein levels remained unchanged at 6 months. This was also the case for α3 and α5 subunits, which are primarily expressed in the hippocampus and are involved in hippocampus-dependent learning and memory^71,72^. We observed an increase of α2 subunit, which could explain the increased unconditioned anxiety detected in hAPP_wt_ mice, a process mediated by α2 containing GABA_A_Rs^68,73^. GABA_A_R-mediated responses are also determined by the intracellular Cl^−^ concentration ([Cl^−^]_i_). Cation-chloride transporters such as KCC2 and NKCC1 are involved in [Cl^−^]_i_ regulation and imbalance in their expression or function can disrupt GABAergic inhibition^34,35^. We previously showed that overexpression of APP in vitro decreases KCC2 expression leading to decreased inhibitory function of GABA^37^. In contrast, Ts65Dn mouse model of DS exhibits increased NKCC1 levels leading to the same outcome^15^. There is also an indication that APP actually interacts with KCC2, stabilizing the transporter at the cell surface and ensuring its function^38^. Surprisingly, here we show that protein levels of KCC2 and NKCC1 remained unchanged in hAPP_wt_, suggesting stable [Cl^−^]_i_ levels and a proper GABA shift. Next to fast acting GABA_A_Rs, GABA_B_Rs mediate slow and prolonged inhibitory synaptic signals. Here we demonstrate that their expression was not altered in hAPP_wt_ mice at 6 months of age. The molecular diversity in the GABA_B_Rs arises from two different subunit isoforms GABA_B_R_1a_ and GABA_B_R_1b_. These subunits differ mainly in the presence of a sushi domain in GABA_B_R_1a_, and together with GABA_B_R_2_ form a functional receptor^74^. GABA can act on presynaptic GABA_B_Rs on GABAergic terminals (autoreceptors) inhibiting further release of GABA through feed-forward inhibition. GABA_B_Rs are also located at glutamatergic terminals where they act as heteroreceptors since GABA from active interneurons can spill over to pre- and postsynaptic GABA_B_Rs on excitatory synapses^75^. In addition, GABA_B_Rs can reduce Ca^2+^ permeability of NMDARs in dendritic spines and enhance the Mg^2+^ block of the receptor^76^. Vice versa, activation of NMDARs by glutamate can start a cascade involving CaMKII and 5’ adenosine monophosphate-activated protein kinase (AMPK) which phosphorylates GABA_B_Rs triggering endocytosis of the receptor^77,78^. Our results demonstrate that at 6 months of age, hAPP_wt_ mice present a slight increase in the NMDAR subunit GluN2B, the expression and the distribution of which might interfere with LTP and memory formation^79^. However, the change observed was very modest and Simón and colleagues reported a more important decrease in the expression of glutamatergic receptors and changes in synapse-associated proteins in hAPP_wt_ mice starting at 8 months, indicating that major NMDAR/AMPAR changes occur later and were probably not detectable at 6 months of age^57^. APP is highly expressed in GABAergic interneurons in the mouse hippocampus^40^ with APP forming a complex with GABA_B_Rs, specifically GABA_B_R_1a_ subunit simultaneously regulating bioavailability and localization of APP and GABA_B_Rs^42^. APP deficits can therefore have an impact on presynaptic inhibition due to reduced GABA_B_R_1a_ transport. Furthermore, the formation of APP-GABA_B_R_1a_ complex protects APP from cleavage by BACE1 and therefore reduces Aβ production^42^. We detected a significant production of human sAPPα and to a lesser extent sAPPβ in hippocampal samples obtained from hAPP_wt_ animals. Moreover, sAPPα and sAPPβ contain an extension domain that consequently binds to the sushi domain of GABA_B_R_1a_, a process that decreases the probability of neurotransmitter release^43^. On brain slices of hAPP_wt_ mice, we observed an increased short-term facilitation at the SC-CA1 synapses as well as an increased LTP induced by TBS. The fact that LTP is increased whereas spatial reference memory is impaired may appear surprising as LTP is classically considered as the cellular mechanism that undergoes experience-dependent changes in synaptic connections and information storage. However, such apparent dissociation between LTP and memory has been observed in mice expressing mutant postsynaptic density protein-95^80^, in mice with forebrain specific ablation of *Stim* genes^81^ or after ablation of Giα1^82^. To explain this effect, Pineda and colleagues hypothesized that amplified responsiveness of CA1 postsynaptic neurons to stimuli would saturate LTP and diminish synapse-specific plasticity required for new memory formation^82^. Recently, a study performed by Rice and colleagues demonstrated that sAPPα per se triggers short-term facilitation in hippocampal neurons^43^. Besides, sAPPα enhances LTP and is sufficient to rescue the decrease of LTP observed in APP KO mice^83,84^. The response to the TBS itself is consistent with a decreased GABA release in hAPP_wt_ mice. Binding of sAPPα to presynaptic GABA_B_R_1a_ would diminish GABA release and therefore increase the disinhibitory process observed between the first and the second burst of stimuli during the TBS^85^. The effect of sAPPα (or other APP metabolites) in enhancing LTP and excitability by acting on presynaptic GABA_B_R_1a_ shown in the present study, does not preclude the effect sAPPα recently shown on pre- and/or postsynaptic a7-nAChR receptors^86^. While levels of glutamate remained unchanged at 6 months of age, GABA significantly decreased in hAPP_wt_ mice compared to controls. Simultaneously, we did not observe any changes in GAD65/67 proteins that are responsible for GABA production. In contrast, an increase in GAD65 expression accompanied with elevated GABA levels have been reported in APP KO mice^70^ which is a possible compensatory mechanism accounting for decreased number of GABAergic neurons or alterations in spine density^87,88^. CGPs are common compounds to regulate GABAergic receptors and have been used in Ts65Dn mice to improve LTP (CGP55845)^89^ or to attenuate the sAPPα-mediated reduction of fEPSP frequency in hippocampal neurons (CGP55845, CGP54626)^43^ antagonizing both pre- and postsynaptic GABA_B_Rs. Here, we were able to utilize CGP36216, a specific GABA_B_R antagonist that only acts on presynaptic GABA_B_Rs^54^ and to show that the observed increased excitability in hAPP_wt_ animals was due to decrease in GABA content not glutamate. Administration of CGP36216 was successful in reducing increased excitability in hAPP_wt_ mice during TBS and short-term facilitation. Taken together, disturbances in network synchronization and oscillatory activity are prevalent in AD and DS patients before the onset of neurodegenerative pathology. Our study emphasizes the role of APP in GABAergic transmission in the absence of amyloid plaques and APP mutations. Therefore, enhancing GABAergic inhibition could be a compelling way to prevent early seizures and neuronal overexcitation in affected individuals as well as reinforce the balance between excitatory and inhibitory signaling.

## Materials and methods

### Animals

Mice overexpressing human wild-type APP under the platelet-derived growth factor (PDGF-hAPP_wt_) have been obtained from Charles River France (stock #004,662) and have been described in detail in the past (I5 line)^44,57^. All transgenic mice were heterozygous with respect to the transgene while non-transgenic littermates served as controls. The present study was conducted using male mice at 3 to 24 months of age. Animals were housed and handled according to the Belgian Council on Animal Care guidelines based on protocols approved by the Animal Ethics Committee of the Université catholique de Louvain. All experiments were carried out in compliance with the ARRIVE guidelines.

### Behavior

#### Modified Y-maze

As previously described^90^ the modified Y-maze is commonly used for assessment of spatial and working memory and consists of three identical opaque open arms. In the modified Y-maze test, mice underwent two consecutive trials. During the first trial only two arms were accessible. Mice were placed in the Y-maze and were allowed to explore the two accessible arms during 10 min. In the second trial, after 30 min inter-trial interval, the third arm was opened and the mice placed back into the Y-maze and had access to all the arms during a time period of 5 min. Mice were video tracked (Ethovision 6.1, Noldus; Wageningen, Netherlands) and the time spent in the novel arm versus familiar arms, the latency to enter the novel arm as well as the number of entries into the novel versus familiar arms were recorded.

#### Open field

During the open field test (OF), in two consecutive days animals were allowed to roam free in the OF without any influence of the examiner. Mice were placed in a square area (60 × 60 cm) and video recorded (Ethovision 6.1) for 20 min. During experimental procedure time spent in the center and the frequency to enter the center of the OF were measured.

#### Light–dark test

Unconditioned anxiety was assessed using the light–dark test (LDT) consisting of a cage divided into two sections of equal size by a partition with a door, with one side illuminated while the other side remained dark. Animals were transferred into the testing room 30 min before trial. At the beginning of the trial, mice were placed into the dark chamber (considered to be a safe area) and were allowed to move freely between the chambers for 10 min. Time spent as well as distance travelled in the illuminated chamber was recorded by a video tracking system (Ethovision 6.1).

#### Elevated plus maze

The elevated plus maze test (EPM) was used to assess anxiety in mice and has been described before^91^. Briefly, mice were placed in an elevated plus maze consisting of two opposing open arms (exposed area) and two opposing closed arms (safe area). The time spent in each arm was recorded by a video tracking system (Ethovision 6.1) for a period of 5 min.

#### Morris water maze

The Morris water maze (MWM) test was used to assess spatial learning and memory^92^. The maze consisted of a round pool with a diameter of 113 cm virtually divided into four quadrants (North, South, West, East) filled with 26 °C opaque water. Visual cues were placed around the pool. The platform was located at the center of the North-East quadrant. On the first day of training mice learn to find the visible dark platform, if they are not able to find the platform during a time span of 1 min, they were placed on top of the platform. On the second, third, and forth experimental day the dark visible platform was replaced by an invisible see-through one. Animals were introduced to the MWM from different quadrants with 1 min time to find the invisible platform. The time latency to reach the platform as well as swim speed and the time spent in each quadrant was measured^91^.

### Western blot analysis

Mice were sacrificed by anesthetics overdose given by intraperitoneal (IP) injection. Brains were dissected, mouse hippocampi were taken and snap frozen in liquid nitrogen. Hippocampal sections were dissociated with a pastel and incubated in RIPA buffer (25 mM Tris HCl pH 7.6, 150 mM NaCl, 1% NP-40, 1% sodium deoxycholate, and 0.1% SDS) for 2 h at 4 °C. Lysates were centrifuged at 4 °C, 10,000*g* for 5 min, and supernatants were kept at − 80 °C until use. Protein concentration was determined by bicinchoninic acid protein assay kit (BCA, Pierce) and the absorbance was subsequently measured with NanoDrop spectrophotometer. Samples were denaturized for 10 min at 70 °C in Laemmli buffer (2×), separated on a 10% TGX gel at 200 V for 30–40 min (Biorad) and transferred onto a nitrocellulose membrane (Biorad) for 1 h at 100 V. Protein transfer was verified using Rouge Ponceau and membranes were subsequently rinsed with TBS-T before blocking with 5% non-fat dry milk for 1 h, at room temperature (RT). Membranes were incubated in appropriate primary antibodies (anti-KCC2 1:3000 (Merck), anti-NKCC1 1:1000 (Abcam), anti-β-tubulin 1:10,000 (Neuromics), anti-human APP W02 1:2000 (Merk), total APP anti-C-terminus 1:4000 (Sigma Aldrich), anti-soluble APP clone 22C11 1:500 (Merck), anti-GABA_B_R 1:500 (Sigma Aldrich), anti-GABA_A_R α1 1:1000 (Alomone labs), anti-GABA_A_R α2 1:1000 (Abcam), anti-GABA_A_R α3 1:1000 (Alomone labs), anti-GABA_A_R α5 1:1000 (Alomone labs), anti-GluA1 1:500 (Merck), anti-GluA2 1:1000 (Merck), anti-GluN2A 1:250 (Merck), anti-GluN2B 1:500 (BD Biosciences), anti-GAD65/67 1:1000 (Abcam)) at 4 °C, overnight. On the second day membranes were rinsed in TBS-T and incubated in adequate secondary antibodies (anti-rabbit (Dako/P448) or anti-mouse (Cell signaling) 1:10,000) for 1 h, RT. Before protein detection with ECL (BioRad) membranes were rinsed with TBS-T (3 × 10 min) and revealed on hyper film (GE Healthcare). Quantification of protein levels was performed by densitometry and reported to β-tubulin expression.

### Organotypic brain slice culture

For the determination of soluble mouse APP, mice were rapidly sacrificed, and their brains taken and transferred into ice-cold artificial cerebrospinal fluid (ACSF) composed of 126 mM NaCl, 3 mM KCl, 2.4 mM CaCl_2_, 1.3 mM MgCl_2_, 1.24 mM NaH_2_PO_4_, 26 mM NaHCO_3_, and 10 mM glucose (bubbled with 95%O_2_-5% CO_2_%). After the removal of the cerebellum and frontal cortex, brains were mounted onto a vibratome and sagittal sections of 150 µm were cut in ice-cold ACSF to obtain the dorsal hippocampus. Brain slices were transferred into a 12-well plate (four brain slices for each animal in one well) filled with ACSF and kept at 37 °C and 5% CO_2_. After an incubation period of 2 h, media were collected and kept at − 80 °C until use.

### Electrochemiluminescence (ECLIA) and enzyme-linked immunosorbent assay (ELISA)

Animals were sacrificed at desirable age by anesthetics overdose, hippocampi were taken and kept frozen at − 80 °C until use. Electrochemiluminescence (ECLIA) assays for human-specific soluble APP (sAPPα/sAPPβ; Mesoscale) and amyloid beta (Aβ_40_/Aβ_42_; Mesoscale) as well as enzyme-linked immunosorbent assay (ELISA) for Glutamate (Abcam) and GABA (Cloud-Clone Corporation) were performed according to the manufacturer’s protocol. Hippocampal tissue was lysed in RIPA buffer, the extracted supernatant was used for quantification and normalized to protein content.

### Electrophysiology

#### Brain slice preparation

Animals were sacrificed by cervical dislocation, their brains harvested and transferred to ice-cold ACSF composed of 126 mM NaCl, 3 mM KCl, 2.4 mM CaCl_2_, 1.3 mM MgCl_2_, 1.24 mM NaH_2_PO_4_, 26 mM NaHCO_3_, and 10 mM glucose (bubbled with 95%O_2_-5% CO_2_%). After the removal of the cerebellum and frontal cortex brains were mounted onto vibratome and sagittal sections of 350 µm were cut in ice-cold ACSF to obtain the dorsal hippocampus. Brain slices were acclimatized in oxygenated ACSF at 32 °C for at least 1 h before use.

#### Field potential recordings

Brain slices were transferred to the recording chamber while continuously being perfused by oxygenated ACSF (2 ml/min) at 30 °C. Field excitatory postsynaptic potentials (fEPSPs) were evoked through a bipolar stimulating electrode which was placed in the Schaffer collaterals (SCs) and recorded by AxoClamp 2B amplifier through a glass electrode which was back-filled with 2 M NaCl and placed in the CA1 region (stratum radiatum). Stimuli consisted of 100 µs pulses of constant currents with intensity adjusted to produce 35% of the maximum response every minute. Responses were stabilized for 30 to 60 min (1 stimulation/min) after placement of the electrodes. All responses were digitalized using Digidata 1322A (Axon Instruments, USA) and recorded on a computer using WinLTP software^93^. Long-term potentiation (LTP) was induced by applying a theta-burst stimulation (TBS) consisting of four trains of five pulses (100 Hz) separated by an interburst interval of 200 ms. fEPSP responses were normalized to the pre-TBS baseline and defined as 100%. Paired-pulse facilitation was calculated as the ratio of the slope from the second to the first response for each paired stimulation event. In a separate experiment baclofen (100 μM) was after LTP induction. Additionally, fEPSP were recorded in response to TBS stimulation at 20 Hz. Basal recordings were obtained during 30 min with 1 stimulation/min. Brain slices were stimulated by one train of five pulses (20 Hz) separated by an interval of 50 ms. In a separate experiment brain slices were additionally constantly perfused with 500 μM CGP36216 (Tocris).

#### EEG electrode implantation

Mice were anesthetized with isoflurane (5% induction, 2% maintenance in O_2_) along with their heads immobilized in a stereotactic frame for implantation of electroencephalography (EEG) electrodes. After exposure of the skull small burr holes were made: one for the epidural ground/reference electrode above the right frontal cortex, one for the tripolar electrode above the right hippocampus and three for the placement of anchor screws (two left and one right parietal). The epidural electrode was custom-made by attaching an insulated copper wire to an anchor screw (Invivo1, 1.75 mm). The tripolar depth electrode was custom made by twisting 3 polyimide-coated stainless-steel wires (California fine wire, 70 μm bare diameter). A distance of 600 µm was maintained between the longest (granule cell layer, DG) and middle (pyramidal cell layer, CA1) electrode tips; 400 µm was maintained between the middle (CA1) and shortest (cortex) tips. This depth electrode was implanted stereotactically (coordinates − 2.0 mm anteroposterior (AP); + 1.5 mm mediolateral (ML) relative to bregma; ± 2.0 mm dorsoventral (DV) relative to dura). Electrophysiological feedback was used during surgery to position the tip of the longest electrode in the granule cell layer of the dentate gyrus upper blade.

#### EEG recordings

Starting one week after implantation mice were connected to the EEG setup which consists of a head stage with a unity gain preamplifier, a 6-channel cable, a commutator, and an amplifier (512×). A data acquisition card (NI, USB-6256, 16-bit, 20 mV input range) digitized signals at 2 kHz and stored them on a PC for offline analysis (Matlab, Mathworks). All recordings were performed under controlled conditions with a 12 h light–dark cycle with lights on between 6AM and 6PM. Room temperature ranged from 20–24 °C with 40–60% humidity. During the EEG recording, mice were housed separately in transparent cages (to allow social interactions) where they were able to move freely. EEG recordings were performed during lights-on periods (12AM-5PM). Recordings were analyzed and epileptiform spikes were manually annotated. Baclofen (Tocris) was administered by intraperitoneal injection (1 mg/kg in 0.9% w/v NaCl).

### Histology

#### Antibody staining on paraffin sections

After sedation animals were perfused transcardially with ice-cold PBS and afterwards with cold 4% paraformaldehyde (PFA). Brains were collected and kept in 4% PFA for at least 2 days at 4 °C before paraffin embedding. Sagittal brain  sections (5 µm) were obtained using a microtome. For histological analysis, only brain sections exhibiting an intact hippocampal morphology were used. Before staining slices were de-paraffinized (xylene, 2 × 10 min) and rehydrated using descending (100–90–70–50%) ethanol (EtOH) concentrations. Antigen retrieval was performed at 95 °C for 10 min using citrate buffer. Cooled down brain sections were blocked with PBS and normal goat serum (NGS) for 20 min, RT. Primary antibody (anti-cleaved caspase-3 1:200, Cell signaling) was diluted accordingly in PBS and 2% bovine serum (BSA) and applied onto brain sections for 2 h, RT. After washing with PBS-T (2 × 5 min) and PBS (1 × 5 min), α-rabbit 1:200 (Vector Laboratories) secondary biotinylated antibody diluted in PBS/NGS was applied for 45 min, RT. Brain sections were incubated using Vectastain ABC kit (45 min, RT) and afterwards washed and incubated in 3,3’-Diaminobenzidine (DAB) for maximum 5 min at RT, the intensity of the reaction was observed using a light microscope. After washing the slides in H_2_O for 10 min, samples were dehydrated with ascending EtOH concentrations (50–70–90–100%) and incubated in xylene (10 min) for final fixation. Dry slides were mounted with DPX and left to dry overnight. Slides were scanned using Slide Scanner SCN400 (Leica) and analyzed with Image Scope software.

#### Nissl staining

Paraffin embedded brain sections were dewaxed, rehydrated (see previous section), and incubated for 20 min in 0.1% Cresyl violet solution. After differentiation of the staining in 70% EtOH brain slices were dehydrated in EtOH, mounted with DPX, and dried overnight. Slides were scanned using the slice scanner SCN400 (Leica) and viewed with Image Scope software. The number of neurons in the CA1 region of the hippocampus was assessed using ImageJ software and cross referenced to the digital Allen Mouse Brain Atlas. For each hippocampus sections of the CA1 area measuring 300 × 300 μm were analyzed. Neurons with round and palely stained nuclei were considered as viable, while shrunken and dark stained cells were considered as dead. Data were expressed as the number of surviving neurons per CA1 section.

### Statistical analysis

Statistical analysis of all data points collected in experiments mentioned in this study was performed using GraphPad Prism 7.03. For each set of experiments “n” represents one brain slice (LTP, short-term facilitation, histology) or one animal (behavior, EEG, western blot, ECLIA/ELISA). Normal distribution of data point was assessed using D’Agostino-Pearson omnibus normality test. For comparison of two groups with normal distributions and equal variances, two-tailed unpaired t-test was performed while for the comparison of two groups with unequal variances a two-tailed unpaired t-test with Welch’s correction was applied. To compare two or more groups with normal distributions and equal variances, a two-way analysis of variance (ANOVA) with Sidak’s post-hoc test was performed. For the assessment of anxiety, the time spent in the center (OF), time spent in the light zone (LDT), the distance travelled (LDT), and the time spent in open arms (EPM) raw data was transformed into a data set expressed as the percentage of the raw data using the Excel PERCENTRANK function. For LTP quantification, experimental groups were compared in the last 15 min of the experiment, when LTP was stable. Experimental datasets are expressed as a mean ± standard error of mean (SEM). Statistical significance was fixed to P ≤ 0.05 (*P ≤ 0.05, **P ≤ 0.01, ***P ≤ 0.001, ****P ≤ 0.0001).

## Supplementary Information

Supplementary Information.
