# Supplementary material for: Overexpression of wild-type human amyloid precursor protein alters GABAergic transmission
Source: Sci Rep. 2021 Sep 2;11:17600. doi: 10.1038/s41598-021-97144-3 (PMC8413381; doi:10.1038/s41598-021-97144-3)

Overexpression of wild-type human amyloid precursor protein alters GABAergic transmission

Anna Kreis[1], Jana Desloovere[2], Nuria Suelves[3], Nathalie Pierrot[3], Xavier Yerna[1], Farah Issa[1], Olivier Schakman[1], Roberta Gualdani[1], Marie de Clippele[1], Nicolas Tajeddine[1], Pascal Kienlen-Campard[3], Robrecht Raedt[2], Jean-Noël Octave[3], Philippe Gailly[1*]

[1]: Université catholique de Louvain, Institute of Neuroscience, Laboratory of Cell Physiology, av. Mounier 53 / B1.53.17, B-1200 Brussels, Belgium.
[2]: Universiteit Gent, Faculty of Medicine and Health Sciences, C. Heymanslaan 10, B-9000 Gent, Belgium
[3]: Université catholique de Louvain, Institute of Neuroscience, Alzheimer Dementia group, av. Mounier 53, B-1200 Brussels, Belgium.
*: Correspondence should be addressed to Philippe Gailly at philippe.gailly@uclouvain.be

Supplementary information

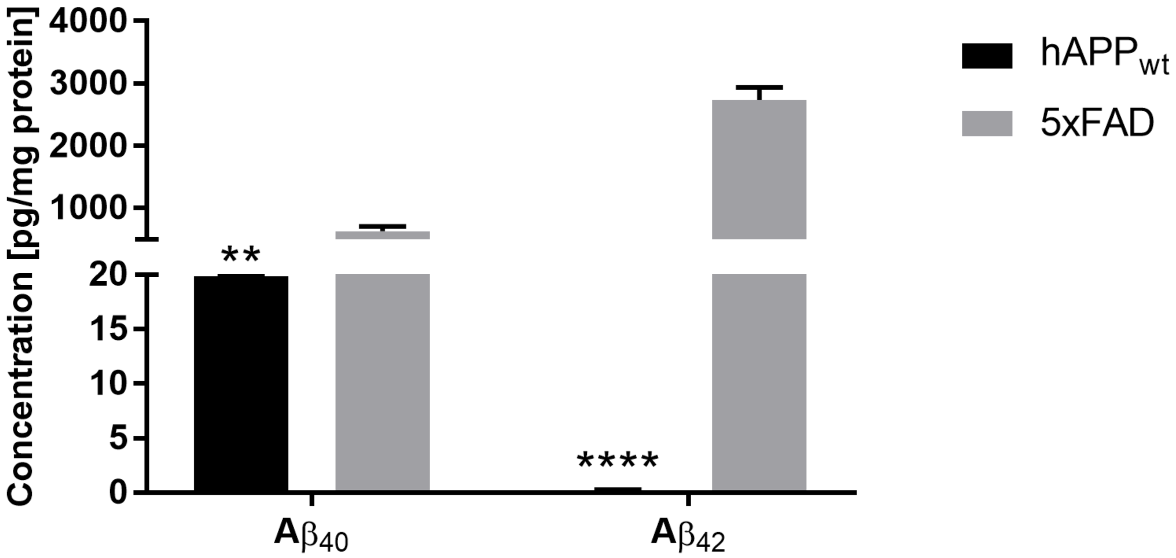

Figure 1: Quantitative determination of amyloid peptides ($A\beta_{40}$/$A\beta_{42}$) in 6 month old male $hAPP_{wt}$ and age-matched 5xFAD mice. Measurement of human $A\beta_{40}$ and $A\beta_{42}$ in hippocampal tissue lysates of $hAPP_{wt}$ (n=3) and 5xFAD (n=3) animals using ECLIA assay, with no detection of amyloid peptides in wild-type control animals. All measurements were normalized to protein content of each sample (Values are means ± SEM; **P≤0.01, ****P≤0.0001, two-way ANOVA with Sidak´s post-hoc test).

Whole western blot scans

Hippocampal lysates were denaturized and separated on a 10% TGX gel (Biorad) and transferred onto a nitrocellulose membrane (Biorad). Protein transfer was verified using Rouge Ponceau (see material and methods). Before blocking with 5% non-fat dry milk, membranes were cut into segments according to the expected molecular weights of the desired protein using a prestained protein ladder (Thermo Scientific/26616) that was visible in the nitrocellulose membrane. For the assessment of soluble APP the SeeBlue protein ladder was used (Thermo Scientific/LC5925) Membrane segments were incubated in the appropriate antibody overnight. On the second day membranes were rinsed and incubated in an adequate secondary antibody. Before the exposure on hyper film, membrane segments were reassembled using the protein ladder as a guideline. Membranes were rinsed after protein detection and stripped for further use with a mild membrane stripping buffer (in 1 L distilled water: 15 g glycine, 1 g SDS, 10 ml Tween 20, pH=2.2).

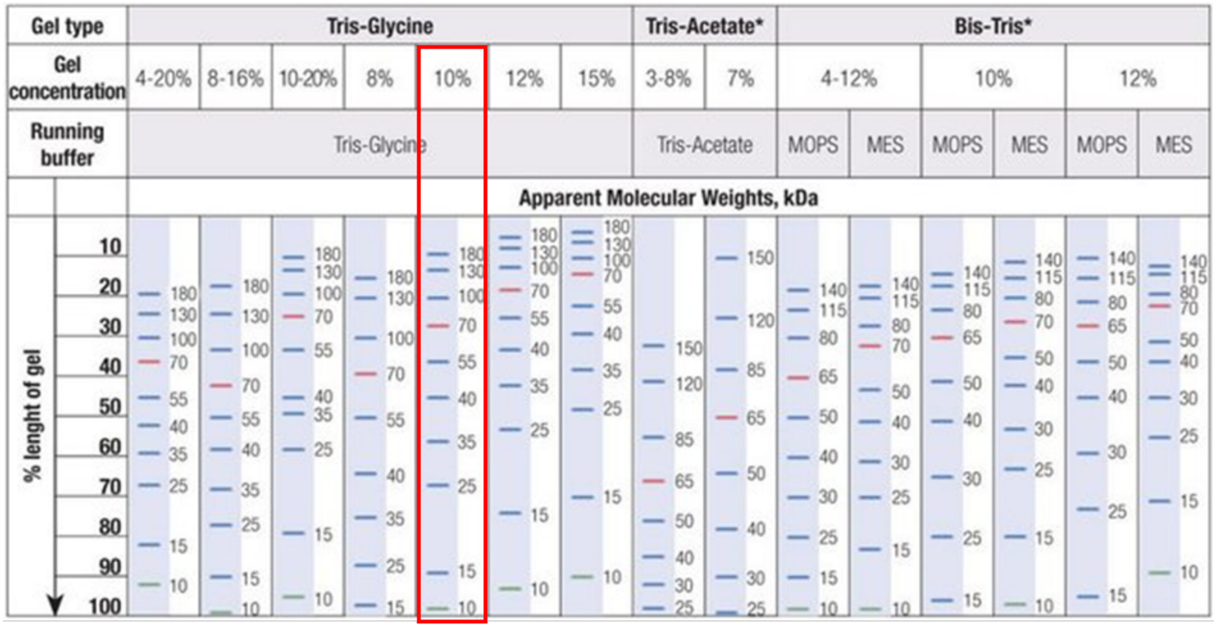

Figure 2: Migration patterns of PageRuler prestained protein ladder (Thermo Scientific/26616) in different electrophoretic conditions. Red box indicates the type of gels used for the western blot experiments with the according protein ladder migration and separation pattern.

Whole western blots

Figure 1 a:

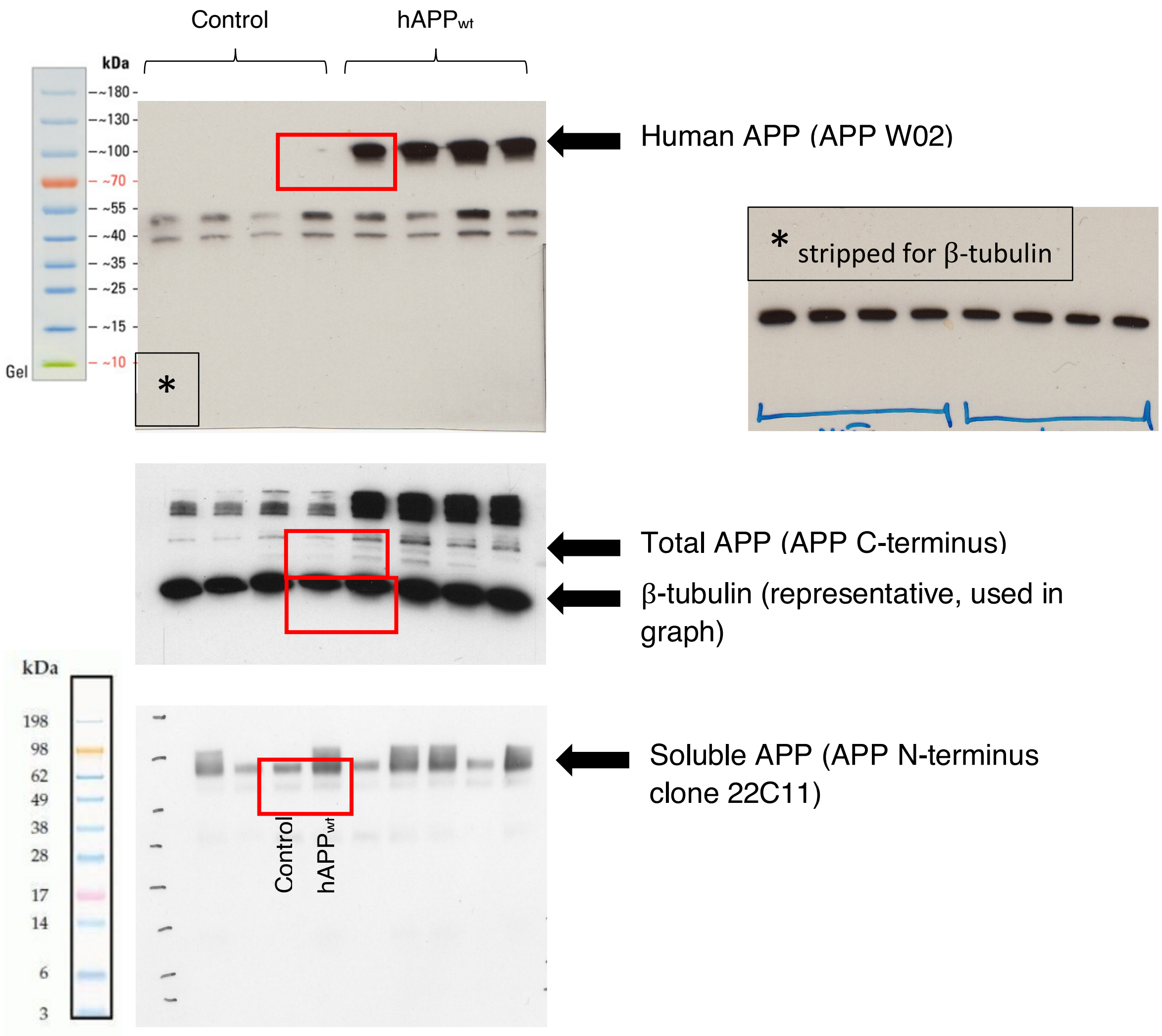

Figure 7 a:

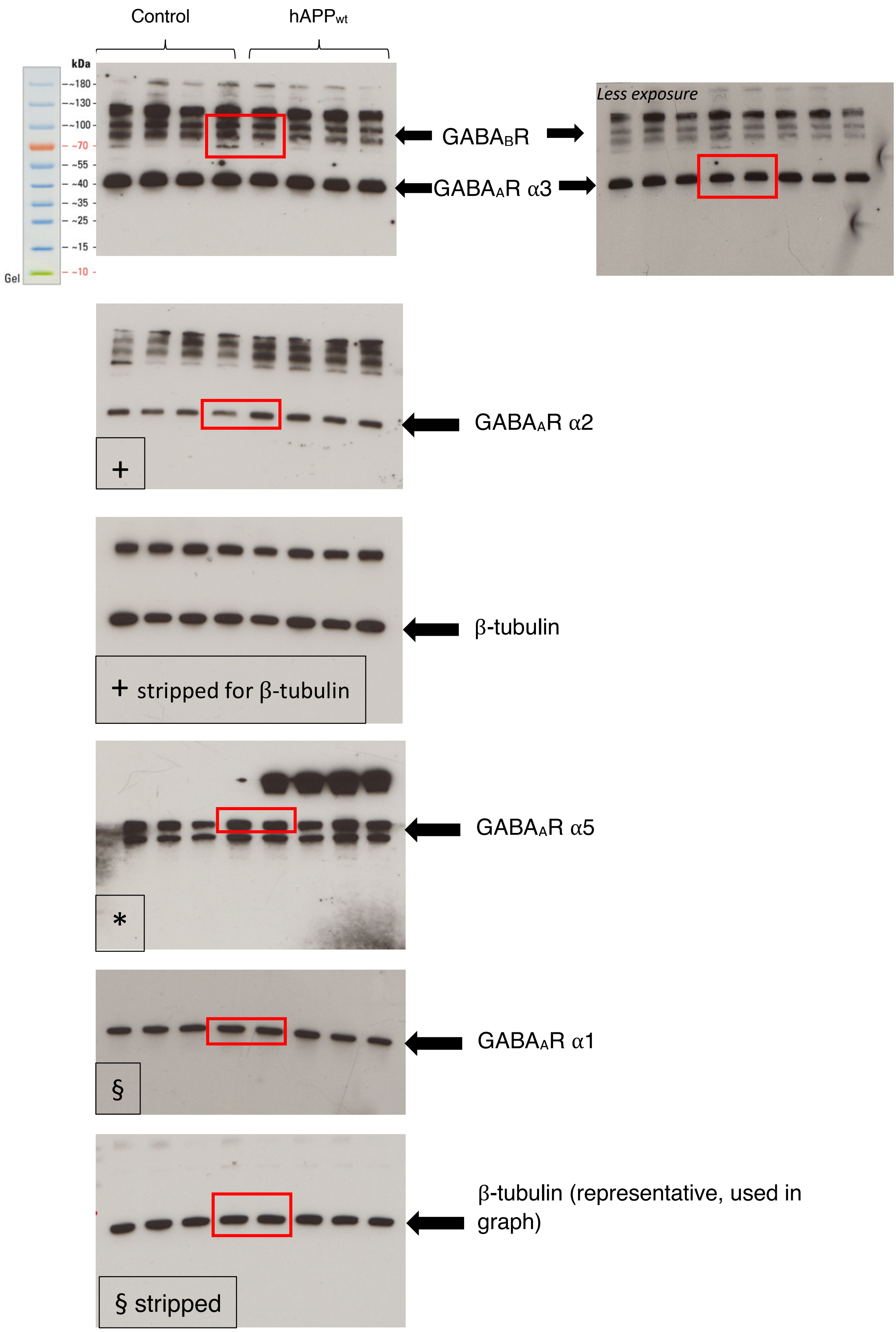
Control
hAPPwt
kDa
~180
~130
~100
~70
~55
~40
~35
~25
~15
~10
Gel
GABABR
GABAAR α3
Less exposure
GABAAR α2
+
β-tubulin
+ stripped for β-tubulin
GABAAR α5
*
GABAAR α1
§
β-tubulin (representative, used in graph)
§ stripped

Figure 7 b:

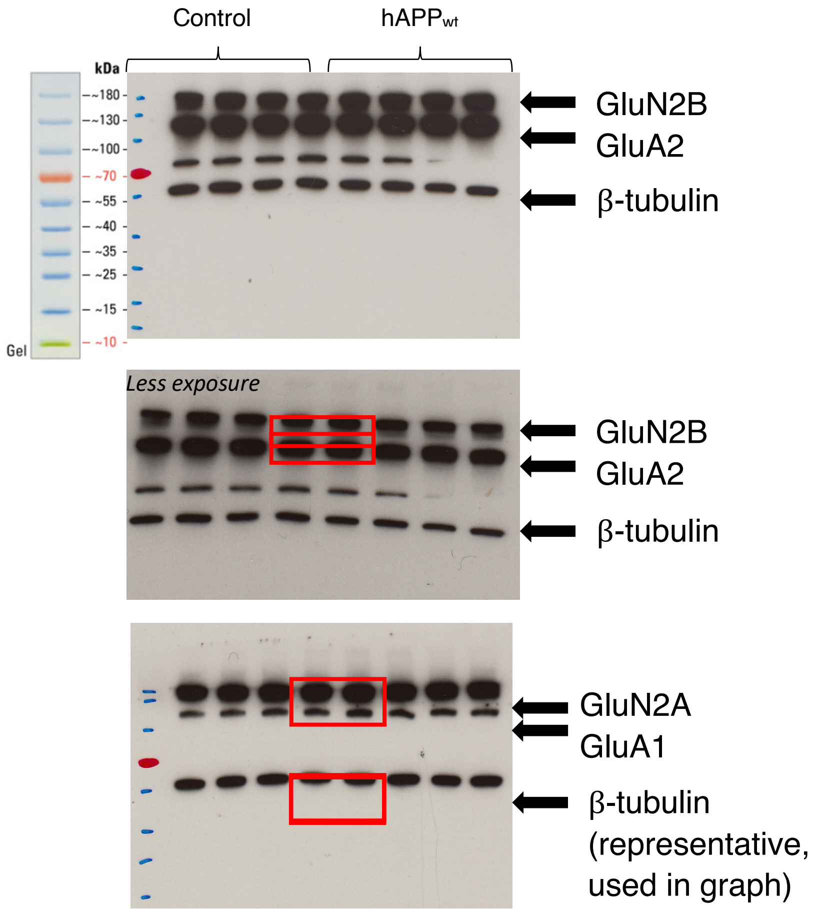
Control
hAPPwt
kDa
~180
~130
~100
~70
~55
~40
~35
~25
~15
~10
Gel
GluN2B
GluA2
β-tubulin
Less exposure
GluN2B
GluA2
β-tubulin
GluN2A
GluA1
β-tubulin
(representative,
used in graph)

Figure 8 c:

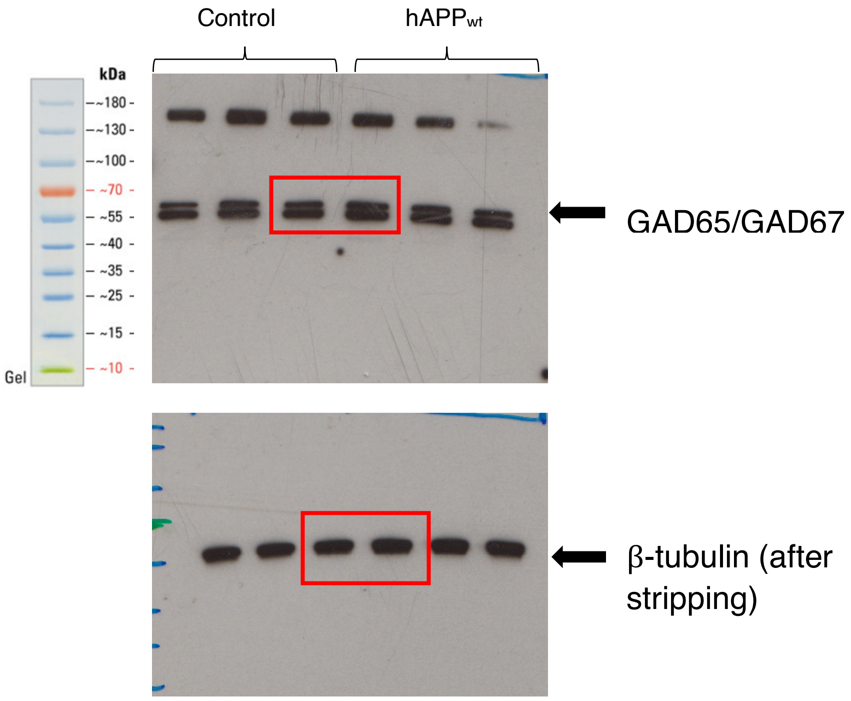

Figure 8 d:

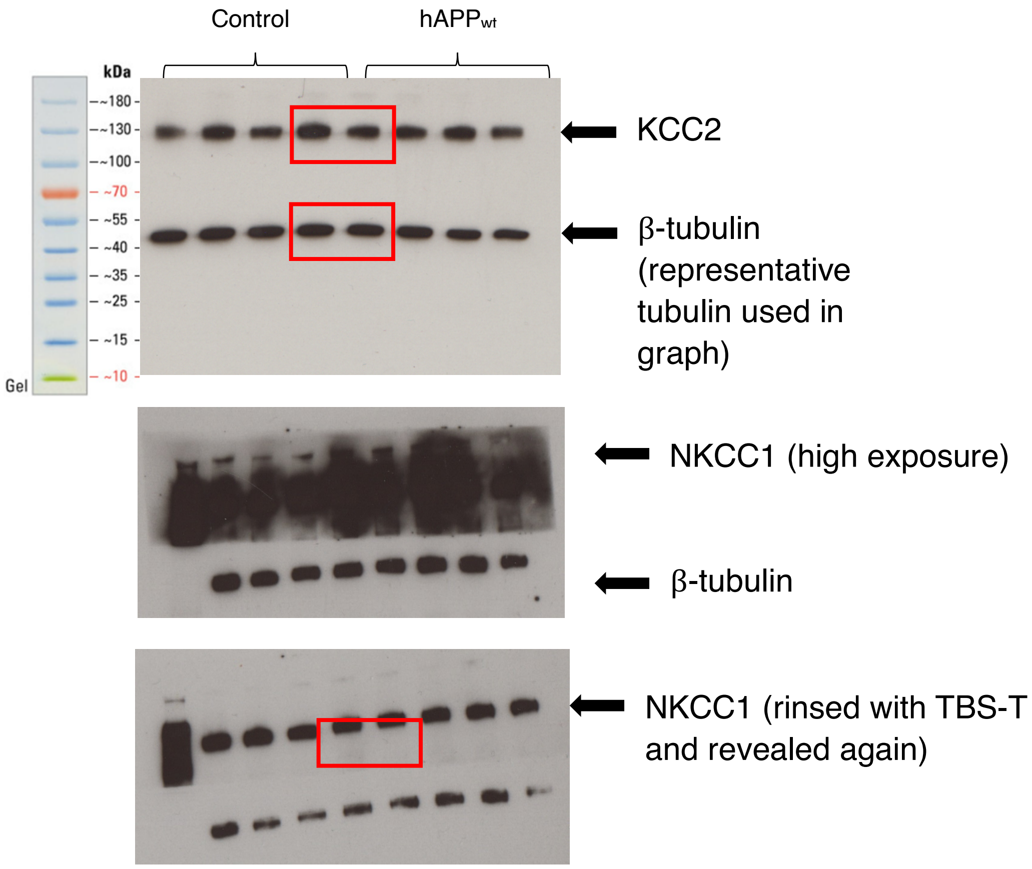

Supplement: Supplementary file 1 — Supplementary Information. [file 41598_2021_97144_MOESM1_ESM.pdf]
